# A versatile NIR probe for multifunctional detection of tumors, fatty liver, and liver injury†

**DOI:** 10.1039/d5sc01433f

**Published:** 2025-06-12

**Authors:** Qihang Ding, Jiqiang Liu, Xinyue Zhang, Jun Li, Keith Man-Chung Wong, Amit Sharma, Pengfei Zhang, Quli Fan, Chao Yin, Hui Zhou, Tony D. James, Jong Seung Kim

**Affiliations:** a State Key Laboratory of Flexible Electronics (LoFE), Institute of Advanced Materials (IAM), Nanjing University of Posts & Telecommunications 9 Wenyuan Road Nanjing 210023 China iamhzhou@njupt.edu.cn iamcyin@njupt.edu.cn; b Department of Chemistry, Korea University Seoul 02841 Korea jongskim@korea.ac.kr; c Department of Chemistry, Southern University of Science and Technology 1088 Xueyuan Blvd Shenzhen 518055 China; d Guangdong Key Laboratory of Nanomedicine, Shenzhen Institutes of Advanced Technology, Chinese Academy of Sciences Shenzhen 518055 China; e Amity School of Chemical Sciences, Amity University Punjab Sector 82A Mohali Punjab 140306 India; f Department of Chemistry, University of Bath Bath BA2 7AY UK t.d.james@bath.ac.uk; g School of Chemistry and Chemical Engineering, Henan Normal University Xinxiang 453007 China

## Abstract

Abnormal viscosity, reduced pH, and elevated levels of superoxide anion (O_2_˙^−^) in living cells are often associated with various biological dysfunctions and oxidative stress. Although some studies have reported probes capable of detecting one or two of these biomarkers, achieving simultaneous, rapid, and convenient detection of all three remains a significant challenge. Herein, we present a rhodamine based probe, DM301, which selectively activates fluorescence in three distinct channels in response to pH, viscosity, and O_2_˙^−^ respectively. Systematic spectroscopic analyses demonstrated DM301's exceptional response to these biomarkers, while *in vitro* experiments confirmed its mitochondrial specificity at the cellular level. To validate the diagnostic potential of DM301, we employed various disease models, including 4T1 tumors, fatty liver, and drug-induced liver injury, all associated with these abnormal biomarkers. *In vivo*, experiments further established the safety of DM301 and demonstrated its specificity to pH, viscosity, and O_2_˙^−^ across different pathological conditions in living organisms. We anticipate that these findings will offer practical and effective strategies for investigating the physiological roles of biomarkers and analytes in diverse biological systems.

## Abstract 2

DM301 is a simple, mitochondria-targeting probe enabling real-time imaging of pH, viscosity, and O_2_˙^−^, with demonstrated diagnostic utility in tumor, hepatic injury, and fatty liver disease models.

## Graphical abstract

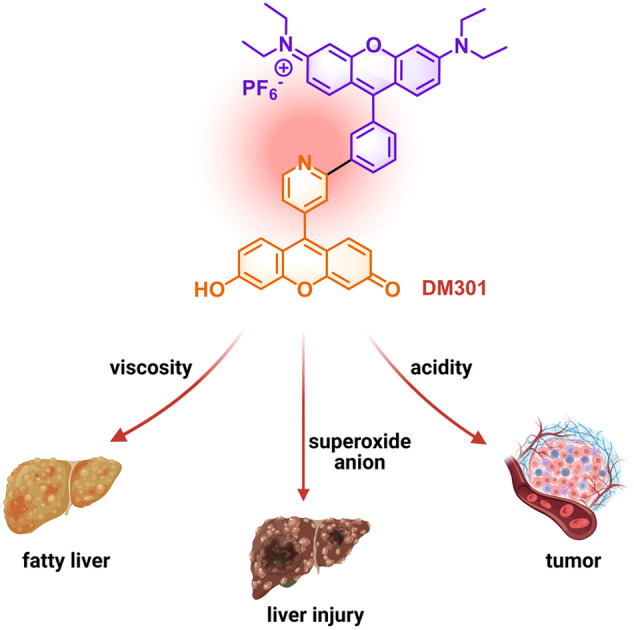

## Introduction

The concentrations of various bioactive substances are tightly regulated to maintain homeostasis in normal cells and tissues.^1^ However, abnormal levels of certain biomarkers, including enzymes, lipids, pH, viscosity, reactive oxygen species (ROS), and reactive nitrogen species, are often associated with diseases and pathological processes, leading to significant fluctuations in these molecules.^2^ For example, cancer is marked by an acidic tumor microenvironment, non-alcoholic fatty liver disease is linked to increased viscosity, and superoxide anion (O_2_˙^−^), an important ROS, is implicated in liver injury.^3^ Since pH, viscosity, and superoxide anion are three critical indicators of various diseases and cellular dysfunction, precise detection of these biomarkers is crucial for unraveling disease mechanisms and identifying potential therapeutic targets.^4^ Fluorescence imaging has become a widely used technique for visualizing bioactive molecules in living organisms, offering high sensitivity, excellent specificity, and low detection limits.^5^ Its superior spatial and temporal resolution enables accurate real-time imaging of these molecules both *in situ* and *in vivo*.^6^ While numerous fluorescent probes have been developed to independently detect pH, viscosity, and O_2_˙^−^, they often face challenges such as limited imaging contrast and an increased risk of false-positive signals in abnormal cells or tissues.^7^ To address these limitations, dual-stimuli responsive probes targeting multiple biomarkers have been developed, offering a more effective approach to enhance imaging resolution and sensitivity both *in vitro* and *in vivo*, thereby improving disease diagnosis. For example, several dual-function fluorescent probes have been designed to simultaneously target two analytes, such as pH and viscosity,^8^ pH and O_2_˙^−^,^9^ and viscosity and O_2_˙^−^.^4*b*^ However, no probe has yet been reported that can simultaneously respond to all three analytes—pH, viscosity, and O_2_˙^−^. There is a pressing need to develop probes capable of responding to these three biomarkers with exceptional sensitivity, selectivity, and visualization capabilities. Such advancements would not only improve the detection of disease biomarkers but also reduce costs by minimizing the number of probes and associated reagents required.

Herein, we engineered a fluorescent probe featuring three spectrally distinct switch-on modes that responds to pH, viscosity, and O_2_˙^−^. The probe molecule is comprised of two fluorescent functional moieties linked by a single covalent bond, imparting viscosity-sensitive characteristics. The fluorescein moiety exhibits sensitivity to both pH and O_2_˙^−^, endowing the probe with the ability to respond to changes in these two factors. Moreover, the probe is designed to target the mitochondria, facilitating near-infrared (NIR) imaging. Targeting the mitochondria enables a more precise investigation of mitochondria-related biological processes and pathological alterations. Systematic spectroscopic analysis confirmed the high sensitivity of the probe under various conditions. Furthermore, cell and animal experiments demonstrated its superior performance in live-cell imaging and various disease models, including 4T1 tumors, fatty liver, and drug-induced liver injury (DILI). This versatile probe holds significant potential for applications in biomedical research and diagnostics.

## Results and discussion

### Synthesis, structural optimization, and multi-responsive fluorescence mechanisms of DM301

**Scheme 1:**
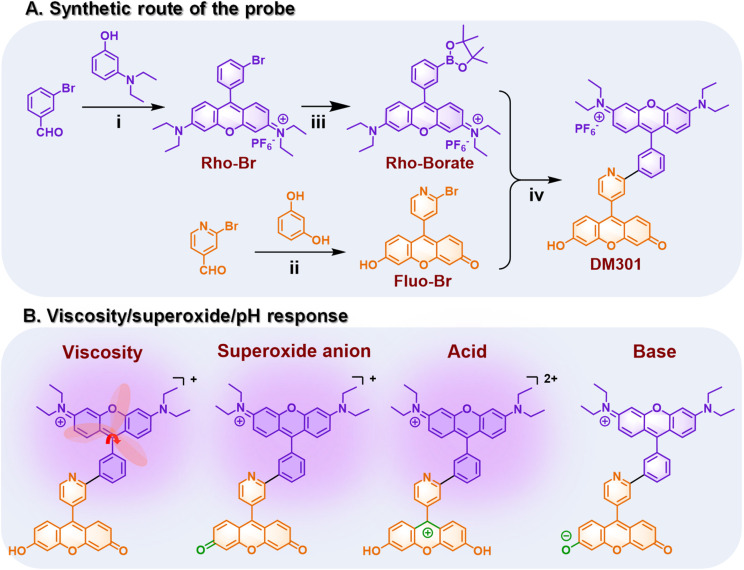
(A) Synthetic route of DM301 (i) 1: *p*-TsOH, AcOH, 70 °C, 10 h. 2: Chlorinil, r.t. 2 h. 3: MeOH, KPF_6_, r.t. 2 h. (ii) Methanesulfonic acid, 140 °C, 16 h. (iii) Anhydrous 1,4-dioxane, bis(pinacolato)diboron, Pd(dppf)Cl_2_ (3.1%), KOAc, 80 °C, overnight. (iv) DMF/H_2_O (3 : 1), Pd(PPh_3_)_4_ (5%), K_2_CO_3_, (16 h) and (B) its proposed response mechanisms toward pH, superoxide (O_2_˙^−^), and viscosity.

**Fig. 1:**
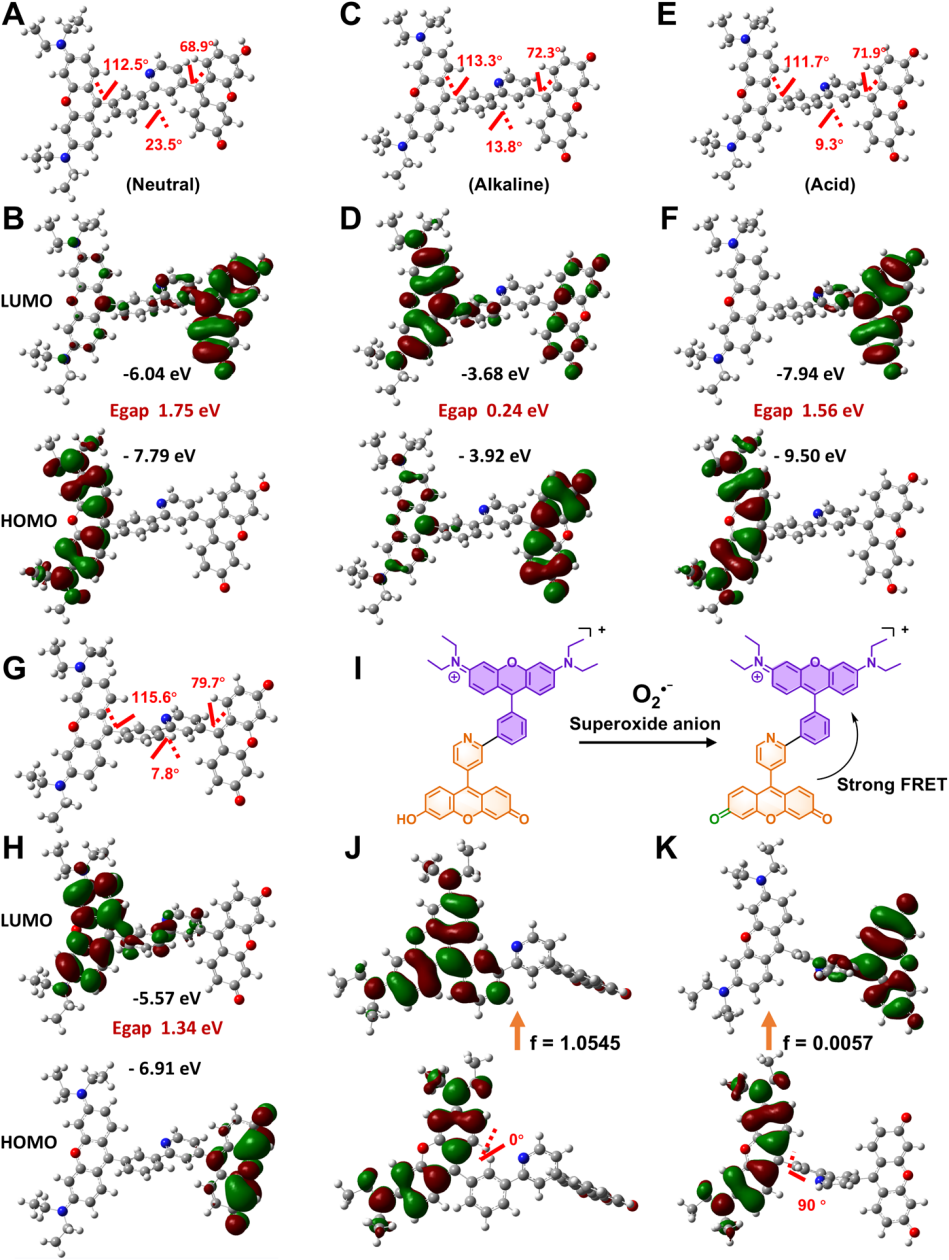
(A–F) The optimized structure and electron density distribution of the frontier molecular orbitals of DM301 in acid, neutral, and alkaline conditions in the ground state, respectively. (G and H) The optimized structure and electron density distribution of the frontier molecular orbitals of DM301 in response to superoxide anion in the ground state. (I) Schematic illustration of the reaction mechanism between DM301 and superoxide anions. (J and K) Frontier molecular orbitals of DM301 in the excited state with dihedral angles of 0° and 90° between the rhodamine moiety and the attached phenyl ring, respectively.

The synthetic strategy for the fluorescent probe DM301 is illustrated in Scheme 1. The rhodamine and fluorescein moieties were synthesized independently. The rhodamine component, Rho-Br, was first converted to a boronate ester *via* Miyaura borylation and subsequently coupled with Fluo-Br using Suzuki cross-coupling. The resulting probe was fully characterized by ^1^H NMR, ^13^C NMR, ^19^F NMR, ^31^P NMR, and HRMS (Fig. S1–S11†). Calculations were conducted to simulate the multi-responsive behavior of DM301. Theoretical calculations of DM301's geometric and electronic structures under acidic and basic conditions (Fig. S12†) indicated that the optimized dihedral angles for C18–C13–C3–C4 (rhodamine/phenyl groups) and C10–C11–C37–C38 (fluorescein/pyridine groups) in both protonated (acidic) and deprotonated (basic) states remained similar to those in the neutral form. However, the C4–C5–C7–N8 angle (phenyl/pyridine groups) was reduced to 13.8° and 9.3° in basic and acidic conditions, respectively, compared to 23.5° in the neutral form. Additionally, the energy gap (Δ*E*) decreased to 0.24 eV (acidic) and 1.56 eV (basic) from the neutral form (1.75 eV), suggesting a strong intramolecular charge transfer (ICT) effect between the rhodamine and fluorescein moieties.^10^ This ICT effect enhances fluorescence and enables the pH response by facilitating the transition between the hydroxyl group and oxygen anion in the fluorescein moiety (Fig. 1).

Similarly, upon interaction with O_2_˙^−^, the optimized dihedral angle of C4–C5–C7–N8 (phenyl/pyridine groups) decreased to 7.8°, (Fig. 1G), compared to 23.5° in the neutral form, further strengthening the ICT effect. The O_2_˙^−^ then oxidized the hydroxyl group (fluorescein moiety) to a ketocarbonyl, thereby enhancing the Förster Resonance Energy Transfer (FRET) process. This reaction decreased the Δ*E* to 1.34 eV, resulting in enhanced, red-shifted fluorescence and increased emission (Fig. 1I and H). To further investigate DM301's response to viscosity, a solvation model density simulation was performed in glycerol. Results revealed that the HOMO–LUMO transition dominated DM301's excitation from S_0_ to S_1_ in a viscous medium. As depicted in Fig. 1J and K, the dihedral angles C18–C13–C3–C4 were set to 0° and 90° to assess fluorescence behavior from the rhodamine moiety. In the orthogonal conformation (90°), LUMO electron density was confined to the fluorescein moiety, while the HOMO was localized in the rhodamine moiety, reflecting charge separation during twisted intramolecular charge transfer (TICT) excitation.^11^ Conversely, in the planar conformation (0°), both HOMO and LUMO were primarily localized on the rhodamine with slight LUMO extension toward the phenyl ring, corresponding to local excitation (LE) and enhanced fluorescence. The calculated oscillator strengths (*f*) were 1.0545 at 0° and 0.0057 at 90°, indicating that restricted rotation in high-viscosity environments limits the TICT state, supporting the observed fluorescence enhancement.

### Viscosity, superoxide, and pH-responsive optical properties of DM301 for versatile biological sensing

**Fig. 2:**
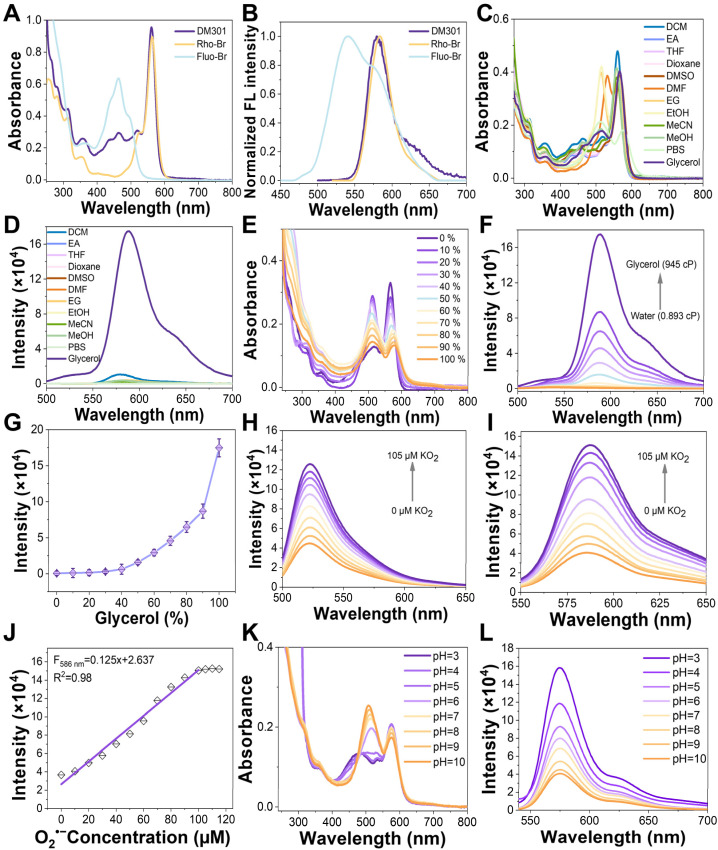
(A) UV-vis absorption and (B) emission spectra of the Fluo-Br, Rho-Br, and DM301 (20 μM) in DCM. (C) The absorption and (D) emission spectra of the probe DM301 (5 μM) were measured in different solvents. (*λ*_ex_ = 480 nm). (E) Absorption and (F) emission spectra of DM301 (5 μM) measured in water/glycerol solutions with increasing viscosity (from 0.893 cP to 945 cP at 25 °C). (G) The fluorescence intensity of DM301 (5 μM) was recorded at 586 nm in different water–glycerol (v : v) ratios. Emission spectra of (H) DHR123 (10 μM) and (I) DM301 (50 μM) measured in DMSO with different concentrations of O_2_˙^−^ (KO_2_ = 0 ∼ 105 μM). (J) Fluorescence intensity of DM301 (50 μM) recorded at 586 nm in response to O_2_˙^−^ levels. (K) Absorption and (L) emission spectra of DM301 measured in Britton–Robinson buffer solution (5 μM) with different pH from 3–10 (5 μM).

Fluo-Br, Rho-Br, and DM301 exhibited maximum absorption wavelengths at 463 nm, 562 nm, and 561 nm, respectively, with corresponding peak emission wavelengths at 530 nm, 575 nm, and 576 nm. Their molar extinction coefficients were determined to be 3.17 × 10^4^, 4.45 × 10^4^, and 4.75 × 10^4^ M^−1^ cm^−1^, respectively (Fig. 2A, B, and Table S1†). Interestingly, DM301 exhibited optical properties derived from its rhodamine moiety through a FRET process, as evidenced by a consistent emission profile under various excitation wavelengths (Fig. S13†). The energy gap of DM301 was comparable to that of Rho-Br (1.79 eV) and significantly smaller than that of Fluo-Br (3.11 eV) (Fig. S14†), aligning with the observed fluorescence emission phenomenon originating solely from the rhodamine moiety.

The optical properties of DM301 were evaluated in various solvents, including CH_3_CN, DMSO, MeOH, EtOH, and glycerol (Fig. 2C and D). While its absorbance remained largely unchanged across solvents, fluorescence emission was significantly enhanced in the highly viscous solvent glycerol, with a quantum yield of 0.104, substantially higher than in other solvents (Table S2†). These results suggest that DM301 exhibits strong potential as a viscosity-responsive probe. To further explore its spectroscopic response to viscosity, DM301 was examined in varying water/glycerol mixtures with different volume ratios. A pronounced red shift in absorption was observed, shifting from 510 nm in pure water to 560 nm in 100% glycerol (Fig. 2E). Additionally, DM301 exhibited weak fluorescence in pure water, but its intensity progressively increased with higher glycerol content (Fig. 2F and G). This effect is likely due to glycerol's high viscosity, restricting molecular rotation in DM301, leading to both a red shift in its absorbance as well as enhanced fluorescence intensity.

We then investigated DM301's response to O_2_˙^−^. O_2_˙^−^ was generated through KO_2_ using a previously reported method^12^ and confirmed *via* the DHR123 assay. A notable increase in the absorption peak of DHR123 at 525 nm was observed with increasing KO_2_ concentrations ranging from 0 to 105 μM, confirming the successful preparation of O_2_˙^−^. Using this method, DM301 was employed for O_2_˙^−^ detection, exhibiting a progressive increase in fluorescence intensity with rising KO_2_ levels (Fig. 2I). A strong linear relationship was observed between the fluorescence intensity at 586 nm and KO_2_ concentrations from 0 to 105 μM, with the intensity plateauing between 105 and 120 μM (Fig. 2J). The limit of detection (LOD) for O_2_˙^−^ was determined to be as low as 124 nM, calculated using the formula 3*σ*/*K*, where *σ* represents the standard deviation of the blank sample and *K* is the slope of the calibration curve.^12^ This result indicates the exceptional sensitivity of DM301 for the *in vitro* detection of O_2_˙^−^. To investigate the pH response of DM301, it was dissolved in Britton–Robinson buffer across a pH range from 3 to 10. As shown in Fig. 2K, the primary absorption peak at approximately 500 nm over a pH range from 7–10 gradually redshifted to 560 nm as the pH decreased. Fitting the pH response to the classical Henderson–Hasselbalch equation yielded an apparent p*K*_a_ value of 6.0 for DM301 (Fig. S15†). Additionally, the fluorescence intensity significantly increased under acidic conditions (Fig. 2L).

To evaluate whether viscosity affects the response of DM301 to O_2_˙^−^, fluorescence measurements were performed in water/glycerol mixtures with varying viscosities (30%, 60%, 90%) in the presence of KO_2_. As shown in Fig. S16,†DM301 exhibited strong and consistent fluorescence enhancement upon KO_2_ addition (∼586 nm) across all viscosities, indicating that superoxide-triggered activation is not hindered by high viscosity. Next, to investigate the combined influence of pH and viscosity on the probe's fluorescence response, DM301 was evaluated in Britton–Robinson buffer solutions with varying glycerol content at pH 3, 7, and 10. As shown in Fig. S17,†DM301 exhibited viscosity-dependent fluorescence enhancement under the three representative pH conditions. Notably, under acidic conditions (pH 3), the sensitivity to glycerol content was markedly higher than that observed at neutral or basic pH, suggesting a possible synergistic effect between protonation and viscosity-induced restriction of intramolecular motion. These results indicate that DM301 is capable of simultaneously responding to both pH and viscosity changes, demonstrating its utility as a multi-stimuli-responsive probe.

To further mimic physiologically relevant conditions where multiple environmental stimuli may coexist, we evaluated the integrated response of DM301 under simultaneous exposure to low pH, high viscosity, and elevated superoxide levels. Specifically, the probe (1 μM) was incubated in a medium containing 40% glycerol (v/v), buffered at pH 4, and supplemented with 35 μM KO_2_ as a superoxide source. As shown in Fig. S18,†DM301 exhibited a significantly enhanced fluorescence intensity at ∼586 nm compared to conditions involving any individual stimulus or dual combinations. This result confirms that DM301 remains responsive and functional under complex microenvironmental conditions, and that its activation mechanisms do not interfere with one another. Such robustness makes DM301 particularly suitable for biological applications where pH fluctuations, redox stress, and viscosity changes often coexist. Furthermore, to assess the selectivity of DM301, we evaluated its fluorescence response toward a series of potential biological interferents, including reactive oxygen species (^1^O_2_, H_2_O_2_, ˙OH, and O_2_˙^−^), proteins (BSA, IgG), metal ions (Ca^2+^, Cu^2+^, Fe^2+^, Fe^3+^), anions (NO_2_^−^, HSO_3_^−^, CN^−^), amino acids (Lys, Gly, Cys), and hypochlorous acid (HClO). As shown in Fig. S19,†DM301 exhibited a pronounced fluorescence increase specifically in response to O_2_˙^−^. While minor increases were observed for ^1^O_2_, H_2_O_2_, ˙OH, and HClO, the signal was significantly weaker. No notable fluorescence changes were detected with the other analytes. These results confirm the high selectivity of DM301 toward superoxide over other biologically relevant species.

Moreover, the stability of DM301 was assessed in PBS and DMEM. As shown in Fig. S20a and b,† no significant changes were observed in the absorption spectra of DM301 under continuous white light irradiation (1.0 W cm^−2^) over 30 minutes, confirming its good photostability in both media. Additionally, long-term stability was confirmed by monitoring the absorption over five days (Fig. S20c and d†), with no noticeable degradation. These results demonstrate that DM301 is stable and photostable under physiologically relevant conditions.

### Mitochondria-targeted and multi-responsive DM301 for visualizing pH, viscosity, and superoxide dynamics in living cells

**Fig. 3:**
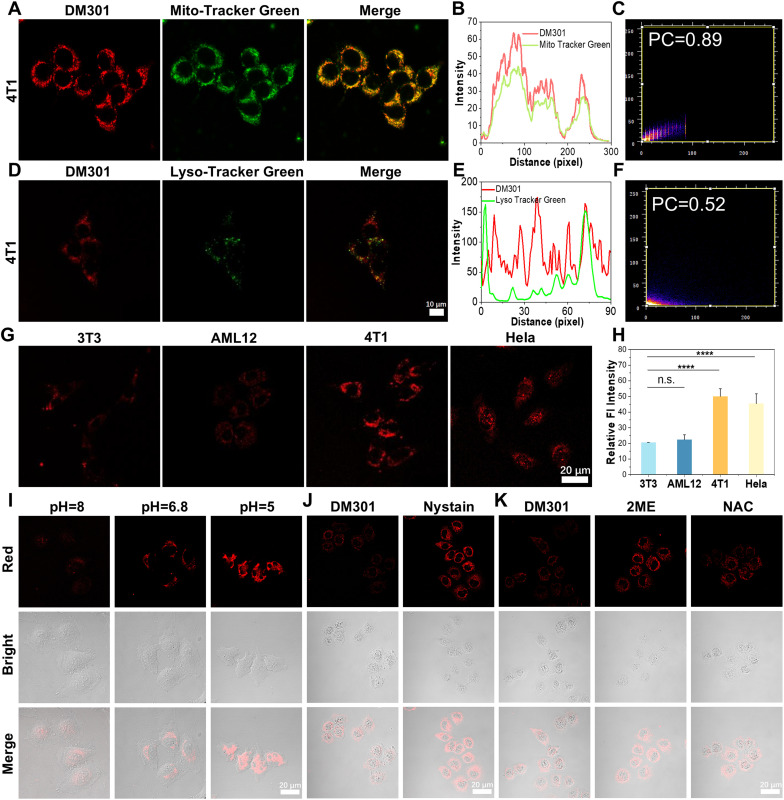
(A) Confocal fluorescence imaging of 4T1 tumor cells stained with DM301 (100 nM) and Mito-Tracker Green. (B) Intensity distribution plots and (C) Pearson's coefficients for co-staining of Mito-Tracker Green and DM301. (D) Confocal fluorescence imaging of 4T1 tumor cells stained with DM301 (100 nM) and Lyso-Tracker Green (scale bar: 10 μm). (E) Intensity distribution plots and (F) Pearson's coefficients for co-staining of Lyso-Tracker Green and DM301. (G) Fluorescence images of DM301 (100 nM) cultured different live cells, including normal cells (3T3, AML12) and cancer cells (HeLa, 4T1), (scale bar: 20 μm). (H) Quantitative analysis of relative fluorescence intensity of (G), (statistical significance was calculated *via* two-tailed Student's *t*-test, ****p < 0.0001. *p* > 0.05 not considered statistically significant (n.s.)). (I) Fluorescence imaging of pH in 4T1 tumor cells using DM301. (J) Fluorescence imaging of viscosity and (K) O_2_˙^−^ in AML12 hepatocytes using DM301 (scale bar: 20 μM).

Next, the cytotoxicity of DM301 was assessed using the MTT method. After 24 hours of incubation with DM301 (320 nM), over 80% of both normal (3T3) and cancerous (4T1) cells remained (Fig. S21 and S22†), indicating low cytotoxicity. Since, mitochondria, the energy factories of cells, are crucial for respiration, metabolism, and signaling, developing small-molecule probes targeting mitochondria is vital for detecting physiological changes and enabling early disease diagnosis. The mitochondrial-targeting ability of DM301 was evaluated *via* co-localization experiments with the commercial dye MTR-green in 4T1 and HeLa cells. A strong overlap was observed with Pearson correlation coefficients of approximately 0.89 and 0.90, confirming effective mitochondrial targeting (Fig. 3A–C and S23†). Additionally, co-incubation of DM301 with LysoTracker Green in 4T1, HeLa and 3T3 cells further confirmed that DM301 selectively targeted mitochondria rather than lysosomes (Pearson correlation coefficients are 0.52, 0.51 and 0.4 in 4T1, HeLa, and 3T3 cells, respectively) (Fig. 3D–F, and S24†).

Next, the pH-responsive behavior of DM301 was evaluated in two normal cell lines (3T3 and AML12) and two cancer cell lines (HeLa and 4T1). Both HeLa and 4T1 cells exhibited strong red fluorescence, whereas minimal fluorescence was detected in normal cells (3T3 and AML12) (Fig. 3G and H), likely due to the higher acidity in cancer cells. Notably, the fluorescence was more intense in 4T1 cells than in HeLa cells, consistent with reports indicating greater acidity in 4T1 cells. To simulate acidic and alkaline conditions, 4T1 cells were incubated with buffer solutions at pH 5.0, 6.8, and 8.0 before treatment with DM301. The strongest fluorescence signal was observed at pH 5.0, confirming DM301's effectiveness in visualizing pH fluctuations in mitochondria (Fig. 3I and S25†). To evaluate DM301's effectiveness in detecting viscosity changes, an antifungal drug, nystatin, which is known to disrupt ionic balance, induce mitochondrial dysfunction, and increase intracellular viscosity was used.^13^ After nystatin treatment, AML12 cells incubated with DM301 exhibited a significant increase in red fluorescence compared to the control group, demonstrating DM301's ability to detect endogenously induced viscosity changes in living cells (Fig. 3J and S26†). Finally, the response of DM301 to O_2_˙^−^ was examined in AML12 cells. O_2_˙^−^ production was stimulated using 2-methylestradiol (2-ME), an inhibitor of superoxide dismutase.^12^ After a 1-hour incubation with 2-ME, DM301-treated AML12 cells showed a 2.8-fold increase in red fluorescence compared to the control group. However, when cells were pretreated with *N*-acetylcysteine (NAC), an O_2_˙^−^ scavenger, before 2-ME incubation, the fluorescence intensity significantly decreased (Fig. 3K and S27†). These results confirm that DM301 is an effective tool for real-time visualization and monitoring of endogenous O_2_˙^−^ in hepatocytes. Furthermore, to quantitatively assess the cellular response of DM301 to superoxide anions, we performed flow cytometric analysis using AML12 cells under chemically induced oxidative stress. Cells were divided into four groups: (i) untreated control; (ii) DM301 only (50 nM); (iii) 10 μM 2-ME pre-treatment followed by DM301 incubation; and (iv) 20 μM 2-ME pre-treatment followed by DM301. As shown in Fig. S28,† the fluorescence intensity was significantly increased in 2-ME-treated cells in a dose-dependent manner, with the 20 μM group exhibiting the highest signal. In contrast, the DM301-only group exhibited minimal fluorescence, indicating low background activation in the absence of oxidative stimuli. These results confirm that DM301 is capable of selectively detecting elevated intracellular superoxide levels in live cells.

### *In vivo* evaluation of DM301 for multi-responsive detection of tumor acidity, liver viscosity, and superoxide in disease models

**Fig. 4:**
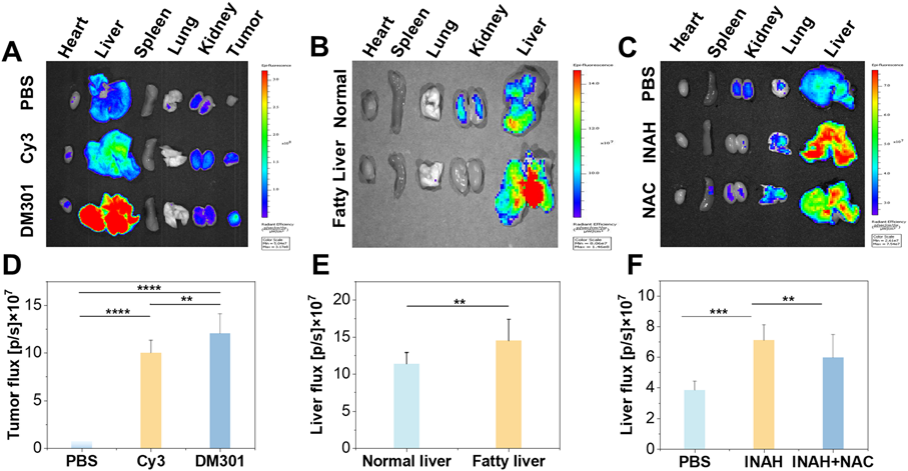
Fluorescence images of major organs in (A) tumor models, (B) fatty liver mice, and (C) liver injury models injected with DM301, compared to control groups. Quantitative analysis of (D) tumors in (A) and (E, F) livers in (B) and (C), respectively (statistical significance was calculated *via* two-tailed Student's *t*-test, **p < 0.01, ***p < 0.001, ****p < 0.0001).

Encouraged by the promising *in vitro* response of DM301 to the three biomarkers, we expanded our investigation to assess the *in vivo* potential across various disease models. To verify DM301's pH response, 4T1 tumor-bearing mice were intravenously injected with PBS (phosphate buffered saline, control), Cy3, or DM301. Cy3, a commonly used cyanine dye with absorption and emission wavelengths similar to DM301, served as a positive control. Organs and tumors were collected and imaged 20 minutes post-injection. The fluorescence intensity in the tumor region of the DM301 treatment group was 1.2 times higher than that of the Cy3 group, indicating the good sensitivity of DM301 for tumor imaging (Fig. 4A and D). Next, we examined DM301's viscosity-sensing capability in a fatty liver model induced by a methionine- and choline-deficient diet over five weeks. In the control group, normal livers appeared bright red, whereas fatty livers in the experimental group showed yellow, uneven surfaces, visually confirming successful establishment of the model (Fig. S29†). This was further validated by H&E staining (Fig. S30†). After injecting DM301 into both groups, *ex vivo* results exhibited significantly stronger fluorescence in the fatty liver group than in controls, indicating higher DM301 accumulation (Fig. 4B and E) in response to elevated viscosity. Finally, DM301 was employed to detect O_2_˙^−^ in DILI models, as oxidative stress and ROS production, including O_2_˙^−^, are hallmark features of DILI. The tuberculosis drug, isoniazid (INAH) was used to induce liver injury, and the establishment of the model was confirmed by H&E staining (Fig. S31†). After 12 hours post-injection of DM301, significant fluorescence was observed in the DILI group compared to the control group (healthy mice without DILI). Notably, pretreatment of DILI mice with NAC, an effective ROS inhibitor, resulted in reduced NIR fluorescence signals, confirming DM301's specificity for O_2_˙^−^ detection (Fig. 4C and F). Collectively, these findings highlight DM301's strong potential for diagnosing diseases like cancer, fatty liver, and liver injury.

## Conclusions

In summary, we have developed a triple-responsive probe, DM301 integrating fluorescein and rhodamine units to sensitively monitor changes in pH, viscosity, and O_2_˙^−^. Theoretical calculations were used to simulate DM301's response to various stimuli, and *in vitro* studies confirmed significant fluorescence changes in response to these different biomarkers. Cellular experiments confirmed DM301's strong mitochondrial targeting and its capability to visualize pH, viscosity, and O_2_˙^−^ fluctuations. Furthermore, DM301 exhibited reliable fluorescent signals for detecting tumors, liver injuries, and fatty liver in mouse models, establishing it as a powerful imaging tool for various diseases. This study offers a promising approach for utilizing a simple, easily synthesized fluorescent probe for precise diagnosis and image-guided therapy.

## Author contributions

Q. D., J. L., and X. Z. equally contributed to this work. All authors discussed the results and commented on the manuscript.

## Conflicts of interest

The authors declare no conflict of interest.

## Supplementary Material

SC-OLF-D5SC01433F-s001

## Data Availability

The data supporting this article have been included as part of the ESI.†
