# Supplementary material for: A versatile NIR probe for multifunctional detection of tumors, fatty liver, and liver injury
Source: Chem Sci. 2025 Jun 12;16(27):12408–15. doi: 10.1039/d5sc01433f (PMC12161149; doi:10.1039/d5sc01433f)
Supplement: SC-016-D5SC01433F-s001 [file SC-016-D5SC01433F-s001.pdf]

Supplementary Information (SI) for Chemical Science.
This journal is © The Royal Society of Chemistry 2025

Supporting Information

A Versatile NIR Probe for Multifunctional Detection of Tumors, Fatty Liver, and Liver Injury

Qihang Ding,[a,b,+] Jiqiang Liu,[c,d,+] Xinyue Zhang,[a,+] Jun Li,[b] Keith Man-Chung Wong,[c] Amit Sharma,[e] Pengfei Zhang,[d] Quli Fan,[a] Chao Yin,[a,*] Hui Zhou,[a,*] Tony D. James,[f,g,*] Jong Seung Kim,[b,*]

[a] Q. Ding, X. Zhang, Prof. C. Yin, Dr. H. Zhou
State Key Laboratory of Flexible Electronics (LoFE) & Institute of Advanced Materials (IAM), Nanjing University of Posts & Telecommunications, 9 Wenyuan Road, Nanjing 210023, China
E-mail: * iamhzhou@njupt.edu.cn; iamcyin@njupt.edu.cn

[b] Q. Ding, J. Li, Prof. J. S. Kim
Department of Chemistry, Korea University, Seoul, 02841, Korea
E-mail: * jongskim@korea.ac.kr

[c] Department of Chemistry, Southern University of Science and Technology, 1088 Xueyuan Blvd., Shenzhen 518055, China

[d] Guangdong Key Laboratory of Nanomedicine, Shenzhen Institutes of Advanced Technology, Chinese Academy of Sciences, Shenzhen 518055, P. R. China.

[e] Amity School of Chemical Sciences, Amity University Punjab, Sector 82A, Mohali, Punjab 140306, India

[f] Department of Chemistry, University of Bath, Bath BA2 7AY, U.K.
E-mail: * t.d.james@bath.ac.uk

[g] School of Chemistry and Chemical Engineering, Henan Normal University, Xinxiang 453007, China.

+ These authors contribute equally to this work.

Experimental Methods
Materials and Characterization

All the solvents and chemicals are commercially available and used as received without further purification. Dulbecco's modified eagle medium (DMEM), and fetal bovine serum (FBS) were purchased from Gibco (Australia). Cisplatin, oxaliplatin, 3-(4, 5-dimethylthiazol-2-yl)-2, 5-diphenyltetrazolium bromide (MTT), LysoTracker Green and Mito-Tracker Green, were purchased from Thermo Fisher Scientific (USA). Dihydrorhodamine 123 (DHR 123), were purchased from Shanghai Maokang Biotechnology Co., Ltd.Nystatin,2-Methoxyestradiol (2-ME) and N-Acetyl-L-cysteine (N-Acetylcysteine) was purchased from Shanghai Aladdin Biochemical Technology Co., Ltd.

^{1}H, ^{13}C, and NMR spectra were acquired on a Bruker 400 MHz magnetic resonance spectrometer. Data for ^{1}H NMR spectra are reported as follows: chemical shifts are reported as δ in units of parts per million (ppm) relative to chloroform-*d* (δ 7.26, s), acetonitrile-*d3* (δ 1.94, s), methanol-*d4* (δ 3.31, s); multiplicities are reported as s (singlet), d (doublet), t (triplet), q (quartet), dd (doublet of doublets), m (multiplet), or br (broadened); coupling constants are reported as a J value in Hertz (Hz); the number of protons (n) for a given resonance is indicated nH, and based on the spectral integration values. Mass spectra were recorded on a Micromass Quattro II triple-quadrupole mass spectrometer or Synapt G2-Si mass spectrometer using electrospray ionization with a MassLynx operating system (Waters, USA). Absorption spectra were measured on a UV−vis-NIR spectrophotometer (Shimadzu UV-3600, Japan). Fluorescence spectra were measured on a Fluorolog-3 spectrofluorometer (Horiba Jobin Yvon, France).

Synthesis

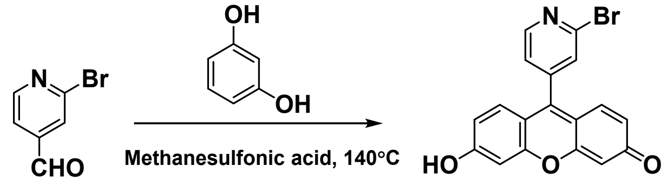

Synthesis of Fluo-Br.

A suspension of 2-Bromo-4-pyridinecarboxaldehyde (500mg, 2.7 mmol) and resorcinol (592 mg, 5.4mmol) dissolved in 15mL $MeSO_3H$. The mixture was heated at 140℃ for 16h with condenser attached. Then the mixture was cooled to r.t. and slowly added into ice with vigorous stirring. The pH of the solution was adjusted to 14 using a solution of saturated NaOH. The aqueous solution was extracted using DCM (100 mL each time) three times. Then the aqueous layer was

extracted using ethyl acetate (100 mL each time) for three times. Then the pH was adjusted to about 6 and an orange precipitate formed. Then the pure product was obtained achieved after vacuum filtration. (211mg, yield 22%). HRMS (MeOH): $C_{18}H_{10}BrNO_3$, theoretical calc. for $[M+H]^+$ m/z =368.19, found $[M+H]^+$ m/z =367.99273. ^{1}H NMR (400 MHz, DMSO-d_6) δ (ppm) = 8.64 (d, J = 4.9 Hz, 1H), 7.89 (s, 1H), 7.61 (dd, J = 4.9, 1.2 Hz, 1H), 7.00 (s, 1H), 6.98(s, 1H), 6.65 (s, 4H). ^{13}C NMR (101 MHz, DMSO-d_6) δ (ppm) = 151.37, 145.40, 144.69, 142.35, 130.53, 128.43, 124.37, 104.04.

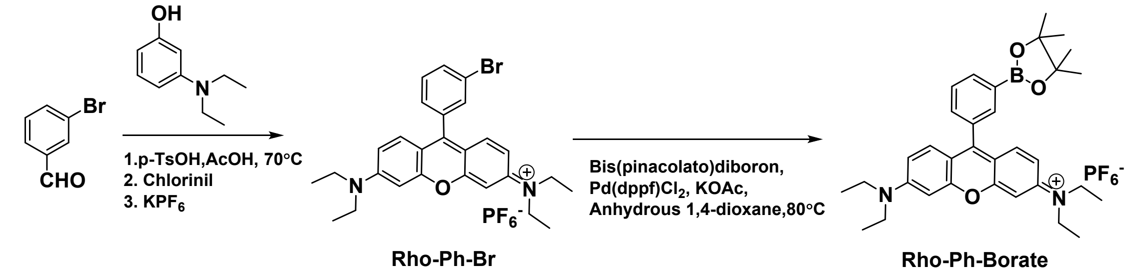

Synthesis of Rho-aryl-Br

A mixture of 3-Bromo-benzaldehyde (500 mg, 2.7 mmol), 3-(diethylamino)phenol (459 mg, 10mmol), p-TsOH (0.0348 g, 0.20 mmol), and acetic acid (20 mL) was heated to 70 °C and stirred for 10 h. The reaction mixture was cooled to r.t., and the pH was adjusted to above 7 with a 10% NaOH solution. The precipitate was filtered and washed with water (50 mL). The solid was dissolved in DCM (50 mL), to which chloranil (0.987 g, 4 mmol) was added. The mixture was stirred for 2 h and then evaporated to dryness. The residue was purified by column chromatography (silica gel; DCM/MeOH, 10:1, v/v) to give a purple solid; yield 347 mg (31.3 %). HRMS (CH_3CN): $C_{27}H_{30}BrN_2O$, theoretical calc. for $[M]^+$ m/z =477.15360, found $[M]^+$ m/z =477.4. ^{1}H NMR (500 MHz, $CDCl_3$-d_3) δ 8.07 (s, 1H), 7.86 (d, J = 3.7 Hz, 2H), 7.45-7.41 (m, 3H), 6.89 (dd, J = 9.5, 2.1 Hz, 2H), 6.85 (d, J = 2.1 Hz, 2H), 3.64 (q, J = 7.1 Hz, 8H), 1.34 (t, J = 7.0 Hz, 12H). ^{13}C NMR (126 MHz, $CDCl_3$-d_3) δ 157.18, 155.80, 155.55, 149.80, 140.16, 137.41, 132.39, 132.09, 129.81, 129.50, 128.70, 127.92, 114.13, 113.28, 96.52, 46.10, 12.56.

Synthesis of Rho-Ph-Borate

The reaction was carried out utilizing the Miyaura borylation reaction, where a solution of Rho-Ph-Br (3.27 g, 17.74 mmol) and pina(colato)dicarbon (3.08 g, 44 mmol) was prepared in DMSO (80 mL). Pd(dppf)Cl_2 (0.817 g, 1.37 mmol) was then added to the mixture as the catalyst to facilitate efficient carbon-boron bond formation. To this mixture, KOAc (0.9 g, 71 mmol) was added to enhance the coupling efficiency and stabilize the reactive intermediates. The reaction mixture was then stirred at 80℃ overnight to ensure complete conversion and optimal product yield. The mixture was hen cooled to r.t. and 100mL water added to the mixture. The mixture was extracted using DCM (50 mL three times) and the organic layer collected and the solvent

removed. The product was used for the next step without further purification.

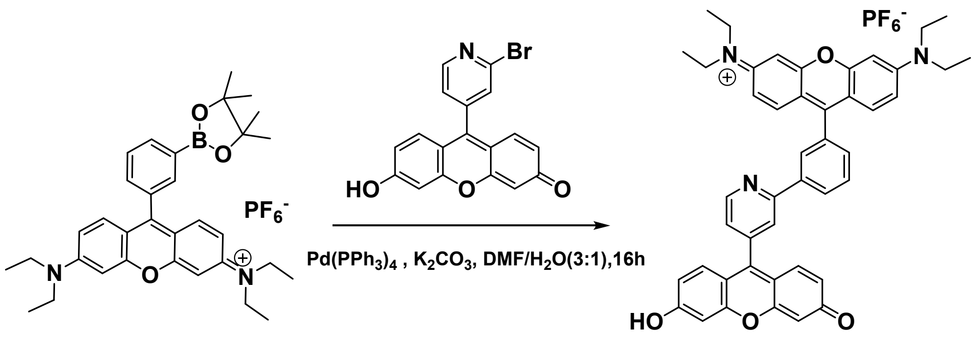

Synthesis of DM301

Utilizing the Suzuki coupling reaction, a solution was prepared by dissolving Rho-Ph-Borate (3.0 g, 16.2 mmol) and Fluo-Br (2.1 g, 16.2 mmol) in DMF (80 mL) to set up the reaction system for efficient cross-coupling. $Pd(PPh_3)_4$ (0.1 g, 0.81 mmol) was then added as the catalyst to promote the formation of carbon-carbon bonds under mild conditions, while K_2CO_3 (4.4 g, 32.4 mmol) served as the base to enhance the reaction efficiency. The reaction mixture was then stirred at 80℃ overnight to ensure complete conversion and achieve the desired product. Then the solvent was removed and the crude product purified by column chromatography on silica gel using DCM:MeOH(20:1) to give the pure product as orange powder (210mg, 17%). HRMS (CH_3CN): theoretical calc. for $[M]^+$ m/z =686.30133, found $[M]^+$ m/z =686.30273. ^{1}H NMR (400 MHz, DMSO-*d$_6$*) δ 8.93 (d, *J* = 3.8 Hz, 1H), 8.52 (d, *J* = 7.2 Hz, 1H), 8.34 (s, 1H), 8.27 (s, 1H), 7.88 – 7.78 (m, 1H), 7.63 (d, *J* = 6.7 Hz, 1H), 7.56 (s, 1H), 7.30 (d, *J* = 9.3 Hz, 2H), 7.14 (d, *J* = 8.9 Hz, 2H), 7.06 (d, *J* = 8.9 Hz, 2H), 6.99 (s, 2H), 6.59 (s, 4H), 3.65 (d, *J* = 5.4 Hz, 8H), 1.21 (s, 12H). ^{13}C NMR (101 MHz, DMSO) δ 157.85, 156.61, 155.65, 155.63, 150.63, 149.98, 146.95, 142.75, 138.72, 133.00, 132.07, 130.90, 129.97, 128.93, 128.71, 128.00, 124.31, 123.87, 123.07, 121.14, 115.03, 113.20, 111.91, 110.10, 107.30, 103.08, 96.50, 45.83, 12.90. ^{19}F NMR (376 MHz, DMSO) δ -69.20, -71.09.

In vitro Experimental Details

UV-Vis absorption and fluorescence spectral studies

Before measurement, the sample was diluted to 20 μM in distilled water (5% DMSO) and equilibrated for 5 min. The UV-Vis-NIR absorbance spectra of **DM301** were recorded on a Shimadzu UV-Vis spectrophotometer. The path length of the cuvette is 10 mm. Emission spectra were collected on a Fluorolog-3 spectrofluorometer (excitation wavelength = 480 nm).

Preparation of $O_2^{\bullet-}$

$O_2^{\bullet-}$ was produced from KO_2 in dry DMSO by an ultrasonic method. The concentration of $O_2^{\bullet-}$ was determined from the absorption at 250 nm (ε = 2682 M^{-1} cm^{-1}).

Fluorescence spectra of DM301 toward $O_2^{\bullet-}$

Different concentrations of $O_2^{\bullet-}$(0-105 μM) were added to **DM301**(20 μM) in distilled water solution (5% DMSO), and each line of the fluorescence spectra in $O_2^{\bullet-}$ channel was recorded after 5 min. λex = 480 nm.

Cell Culture and Cytotoxicity Assay.

The cancer cells (Hela and 4T1) and the normal cells (AML12 and 3T3) were acquired from the Institute of Basic Medical Sciences of the Chinese Academy of Science. DMEM high glucose medium was used to cultivate Hela cells and 4T1 cells. These cancer cells were cultured in the above medium containing 10% fetal bovine serum and 1% antibiotics (penicillin/streptomycin; 100 U mL^{-1}) at 37 °C in a 5% CO_2 atmosphere.4T1 cells and 3T3 cells were inoculated in 96-well plates on average for culture. After 24 h incubation, the medium was replaced by a fresh medium containing different concentrations of **DM301** and incubated for an additional 24 h. Then 20 μL MTT was added (MTT was prepared in advance and the final concentration was 5 mg/mL) and placed in a constant temperature incubator for further culture for 4 h. Then the medium was sucked away and 100 μL dimethyl sulfoxide was added to the 96-well plate, and then the 96-well disk was gently shaken. Finally, the data was determined using microplate reader.

Cellular uptake imaging

HeLa cells, 3T3 cells, AML12 or 4T1 cells were seeded on 12-well plates (2.5 × 10^5 cells in each well) and allowed to adhere overnight. Cells were incubated with 100 nM **DM301** at 37 °C. After being washed three times with PBS, cells were visualized by CLSM imaging.

Intracellular colocalization by fluorescence imaging

HeLa/ 4T1 cells were seeded on confocal dishes (5 × 10^4 cells/dish) and allowed to adhere overnight. Cells were firstly incubated with **DM301** (100 nM) for 6 h and then further incubated with MitoTracker Green (75 nM, 30 min), Lyso Tracker Green (200 nM, 45 min). After being washed three times with PBS, cells were visualized by CLSM imaging. The Pearson correlation coefficient was quantified using ImageJ.

Imaging of viscosity response

AML12 cells were divided into two groups. The control group cells were stained with **DM301** (100 nM) for 6 h. Next group cells were stimulated with nystatin (10 μM) for 30 min, then stained with **DM301** (100 nM) for 6 h. After being washed three times with PBS, the cells were visualized by CLSM imaging.

Imaging of pH response

AML12 cells were divided into three groups. The group 1 cells were treated with buffer (pH=8) for 1 h. The group 2 cells were treated with buffer(pH=6.8) for 1 h. The group 3 cells were treated with buffer (pH=5) for 1 h. Each group was then cultured with **DM301**(100 nM) for 6h. After being washed three times with PBS, cells were visualized by CLSM imaging.

Fluorescence imaging of $O_2^{\bullet -}$ under 2-ME stimulation

AML12 cells were divided into three groups. The control group cells were stained with **DM301** (100 nM) for 6 h. The 2-ME stimulated group cells were incubated with 2-ME (3.0 μg/mL) for 1 h, then stained with **DM301** (100 nM) for 6 h. The 2-ME and NAC stimulated group cells were pretreated with NAC (10 μM) for 2 h, followed by adding 2-ME (3.0 μg/mL) for 1 h and then stained with **DM301** (100 nM) for 6 h. After being washed three times with PBS, cells were visualized by CLSM imaging.

Animals

All animal care and experimental procedures adhered to the Guide for the Care and Use of Laboratory Animals and received approval from the SIAT Animal Care and Use Committee, Chinese Academy of Sciences (Serial number: SYXK (Yue) 2021-0119). Balb/c type mice (5 weeks, male) were purchased from Beijing Vital River Laboratory Animal Technology Co., Ltd. Experiments were conducted according to the National Institute of Health Guide for the Care and Use of Laboratory Animals. Mice were fed with Methionine- and Choline-Deficent Diet (MCD) feed to induce the fatty liver mice model. After induction of 5 weeks, their livers were isolated from the mice.

***In vivo* imaging**

The NAFLD model by feeding BALB/c mice (6-month-old, male) with 60 kcal% high-fat diet (Research Diets) for 8 consecutive weeks. The 4–6-week-old nude mice were chosen and 4T1 tumors were established by s. c. injecting 2× 10^6 4T1 cells. When the tumor volume reached 100 mm^3, the tumor-bearing mice were randomly divided into three groups (PBS, Cy3 and **DM301**, n = 3). Acute liver injury model was established in BALB/c mice (6-month-old, female)

with isoniazid (180mg/kg) or NAC (180mg/kg). Mice were intravenously injected with 100 μL **DM301** (50 μM in PBS containing 5% DMSO as cosolvent , then they were sacrificed, and their isolated organs including tumors were imaged. The fluorescence was triggered at 540 nm and collected at 600 ± 20 nm with a IVIS LUMINA K Series Ⅲ small animal in vivo imaging system.

Statistical analysis

All experiments were performed three times and the data were presented as means ± standard deviation. Statistical analysis was performed according to the Student's t-test and one-way analysis of variance using SPSS software. Differences were considered statistically significant at the following p values: *$p < 0.05$, **$p < 0.01$, and ***$p < 0.001$, ****$p < 0.0001$.

Supporting figures

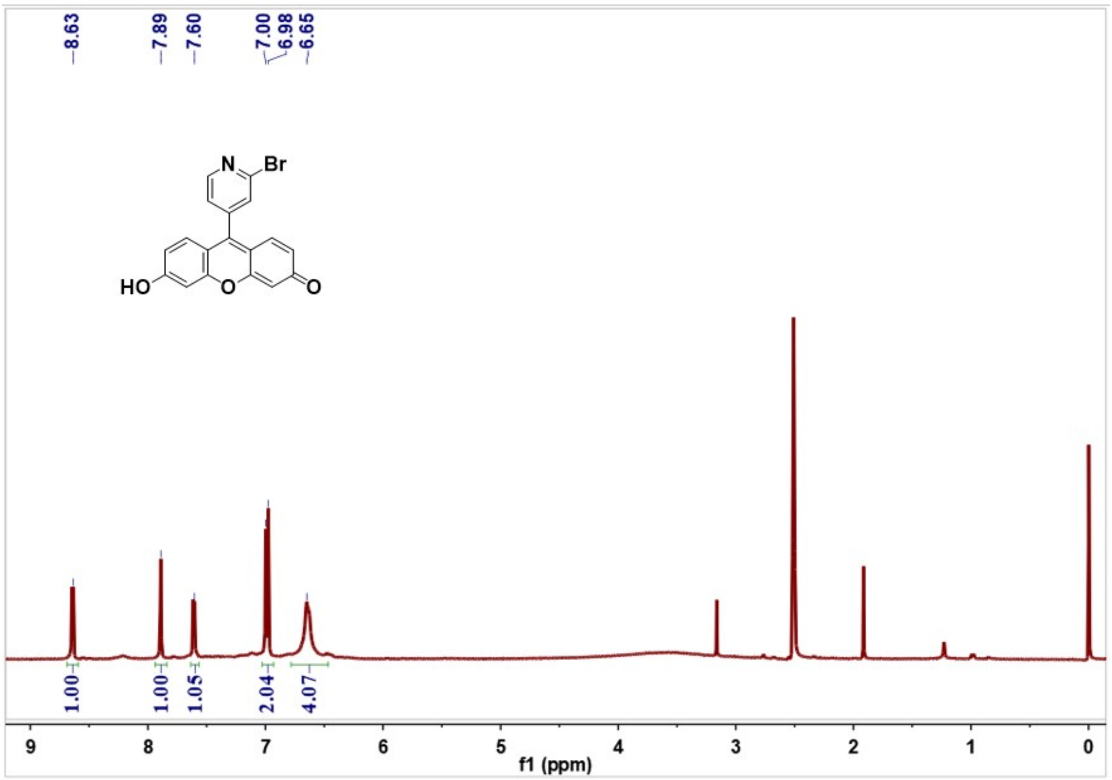

Figure S1. ^{1}H NMR of Fluo-Br in DMSO-d_6

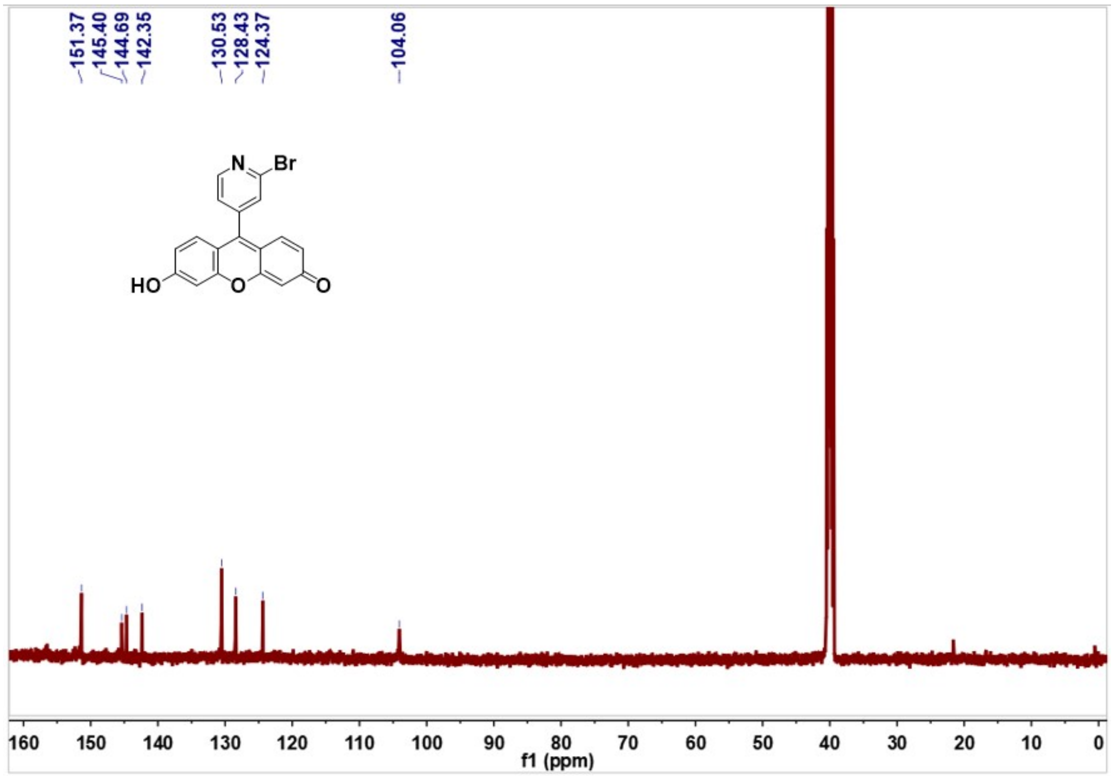

Figure S2. ^{13}C NMR of Fluo-Br in DMSO-d_6

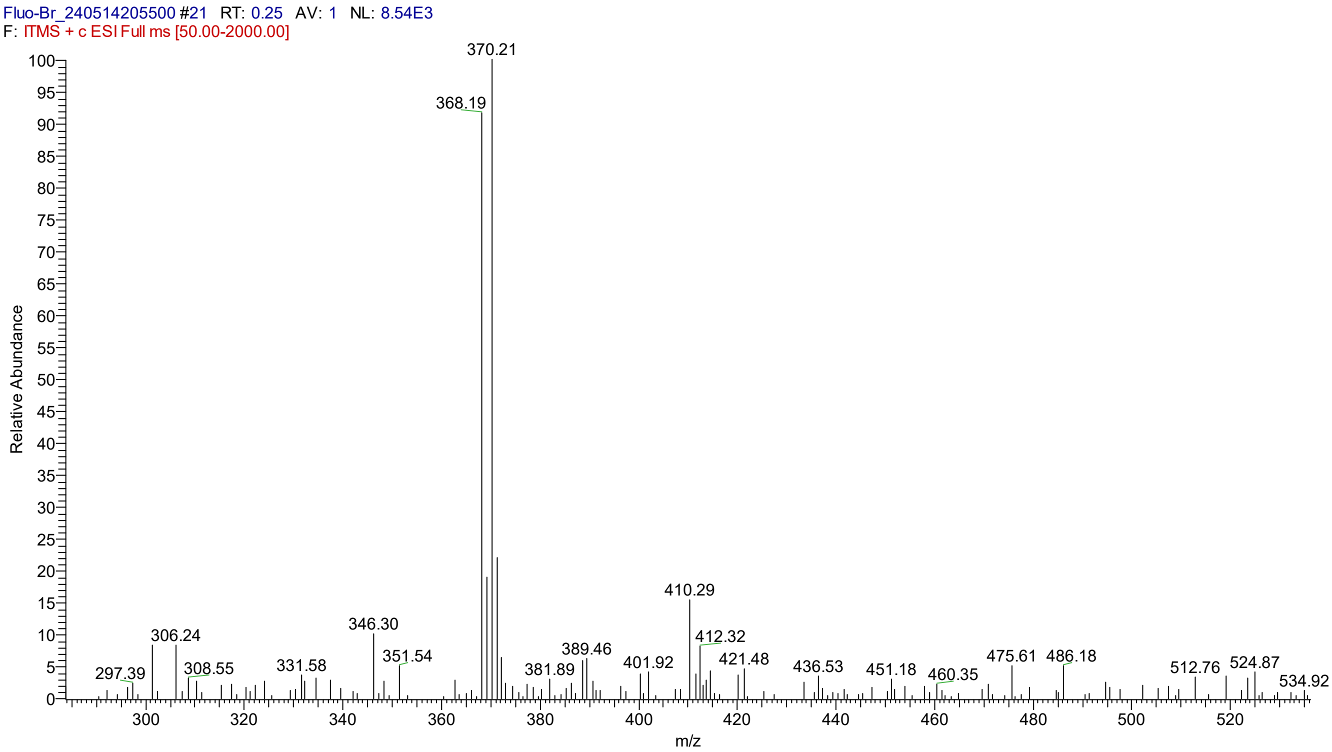

Figure S3. MS spectrum of Fluo-Br. [M+H]+ m/z =367.99, found [M+H]+ m/z =368.19.

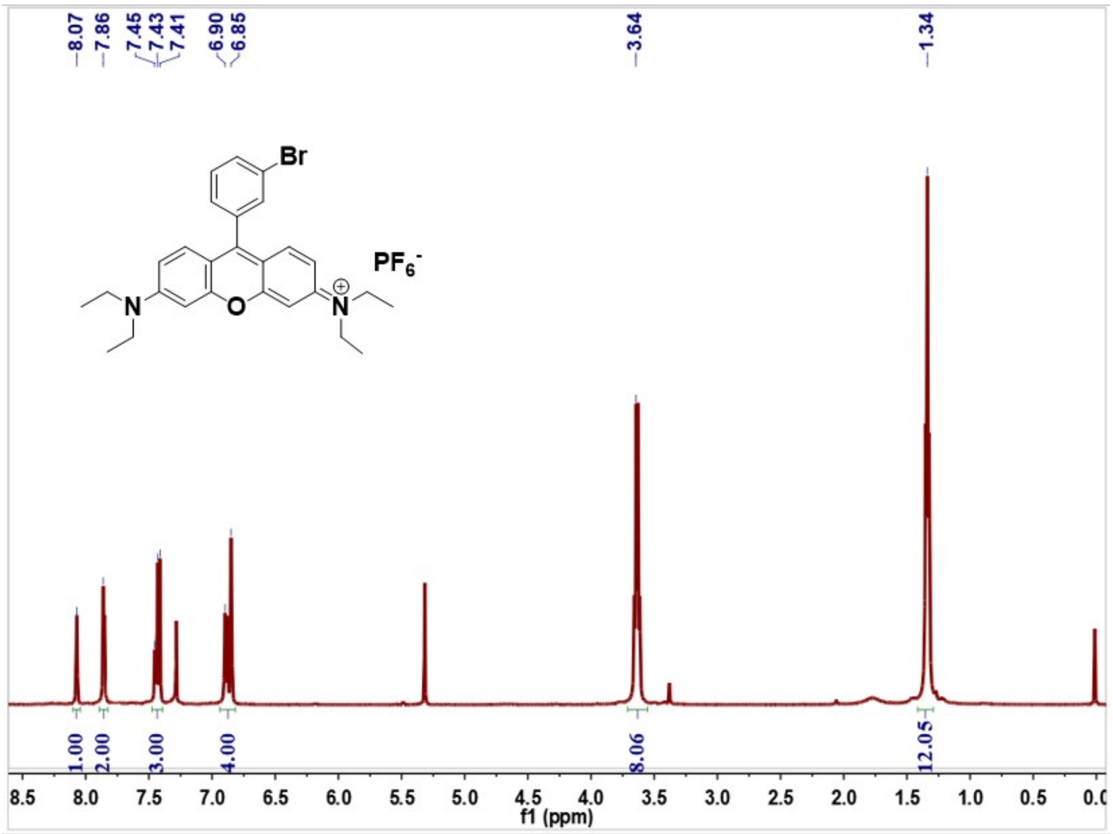

Figure S4. ^{1}H NMR of Rho-Ph-Br in $CDCl_3$-*d*.

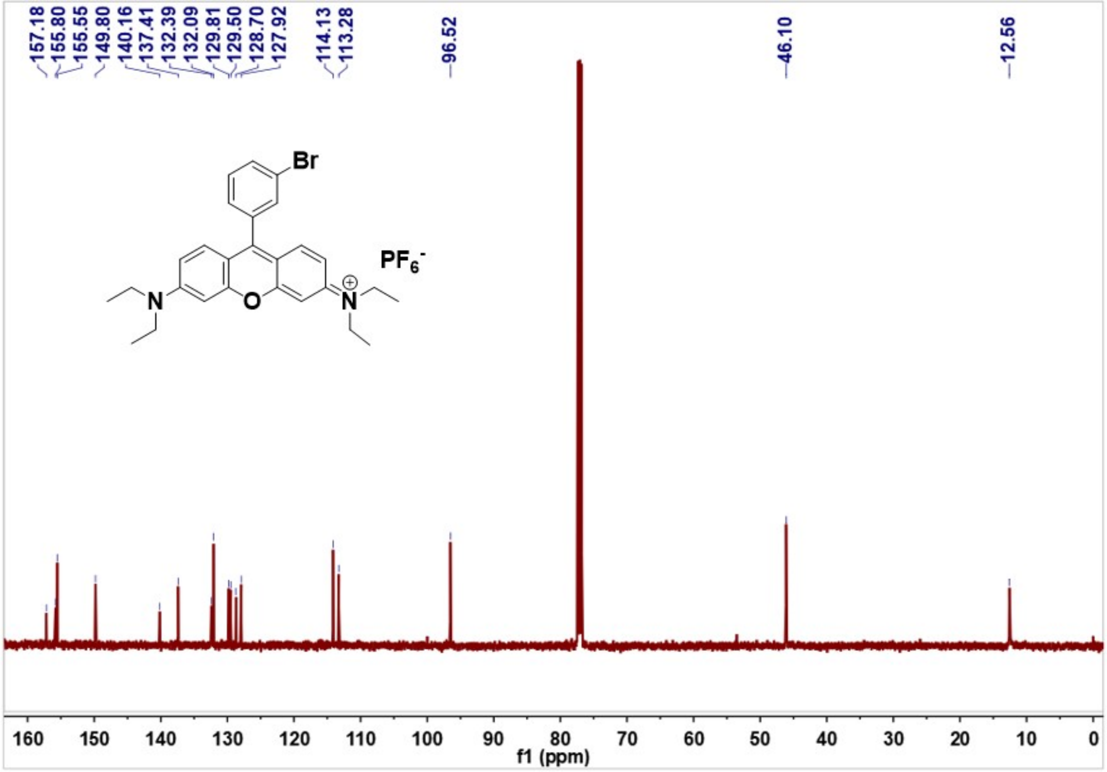

Figure S5. ^{13}C NMR of Rho-Ph-Br in $CDCl_3$-*d*.

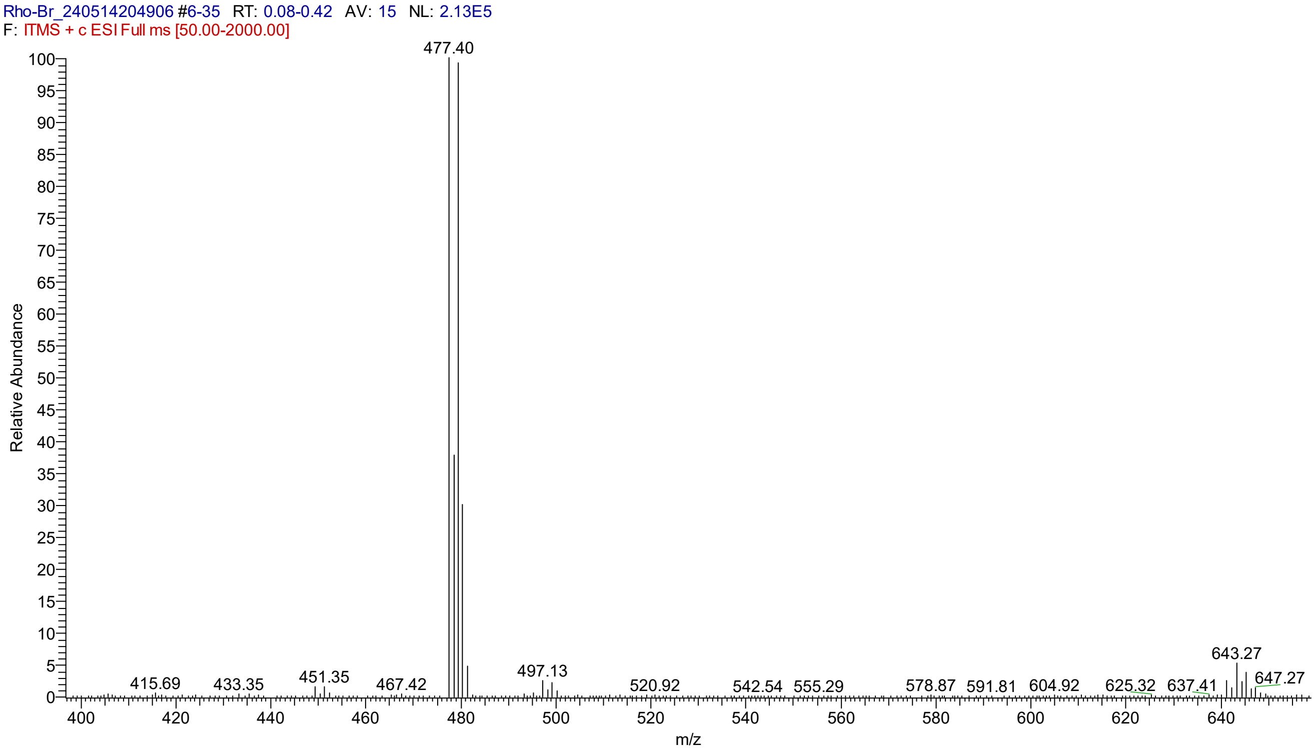

Figure S6. MS spectrum of Rho-Br. Theoretical calc. for [M]+ m/z =477.15, found [M]+ m/z =477.40.

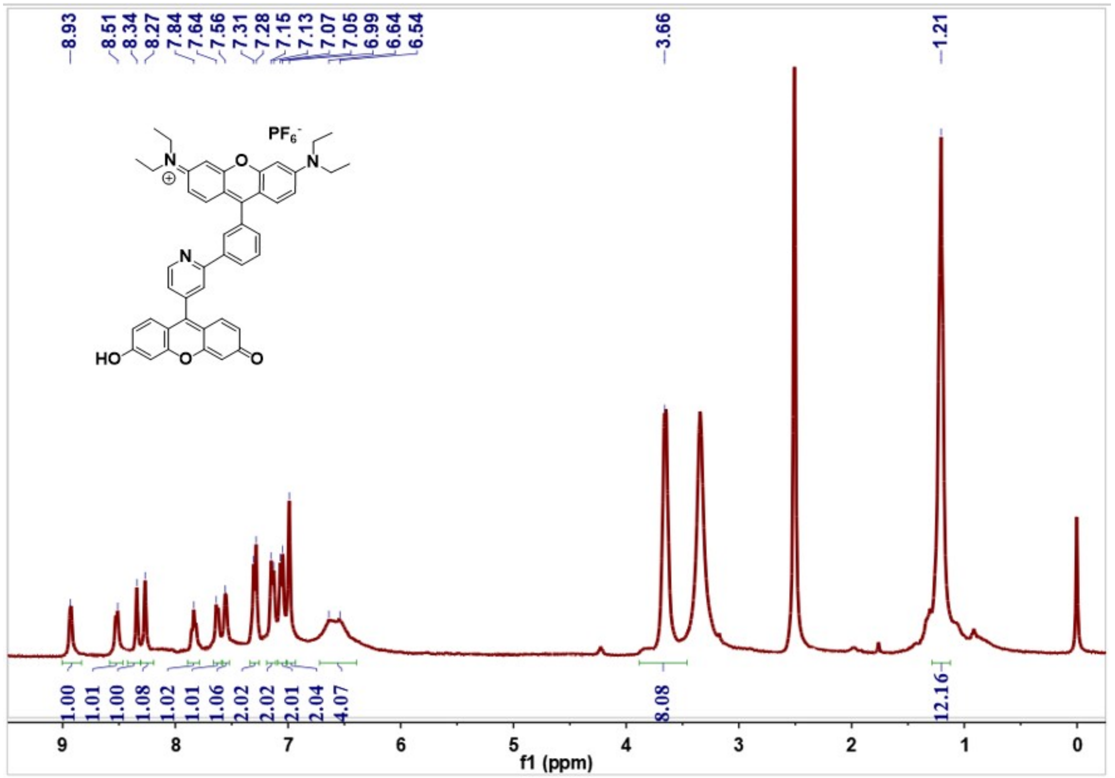

Figure S7. ^{1}H NMR spectrum of **DM301** in DMSO-d_6

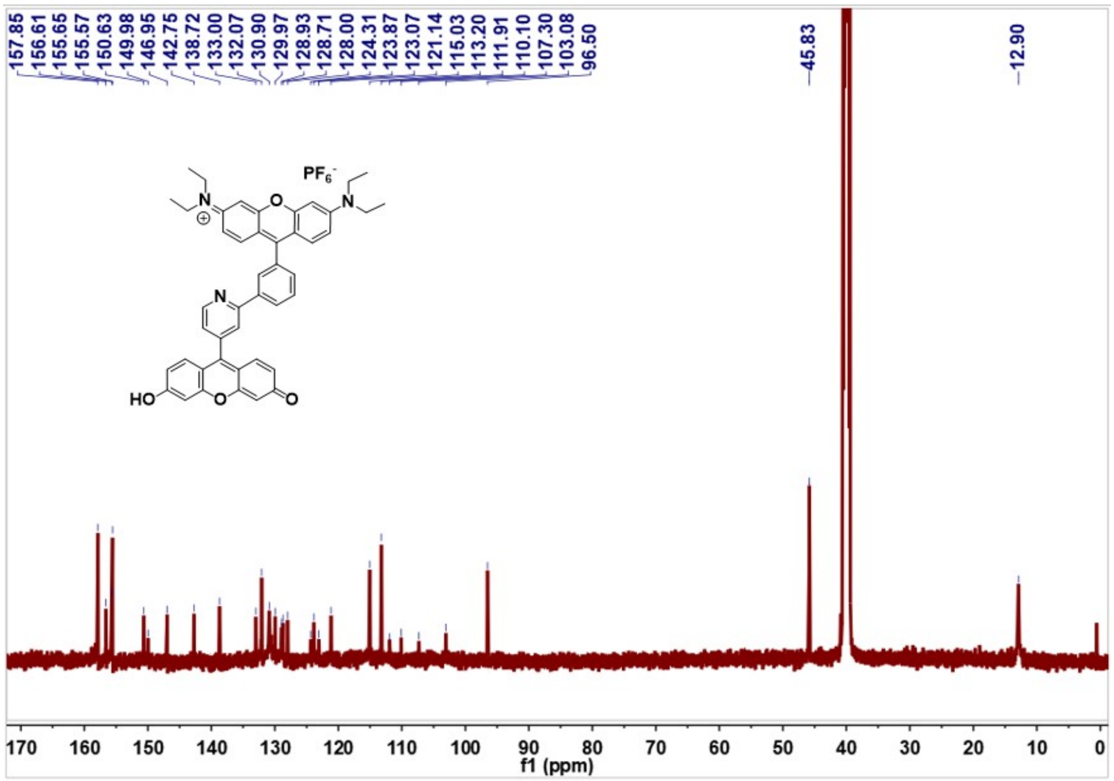

Figure S8. ^{13}C NMR spectrum of **DM301** in DMSO-d_6

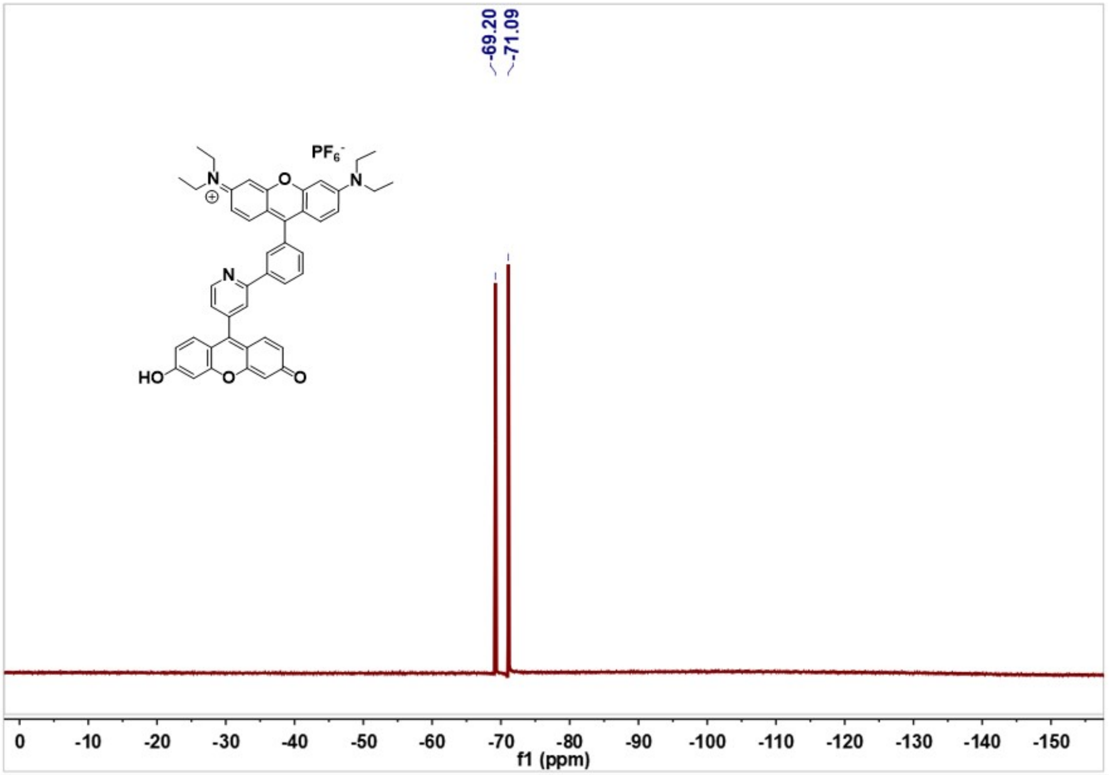

Figure S9. ^{19}F NMR spectrum of **DM301** in DMSO-d_6

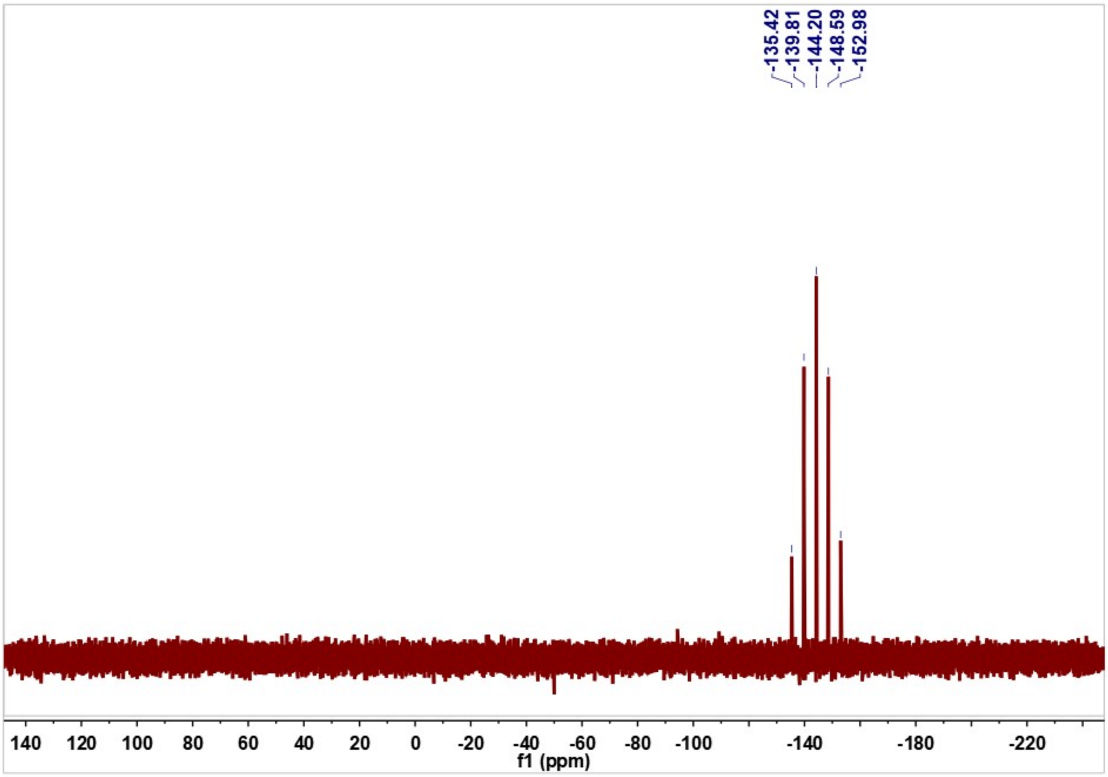

Figure S10. ^{31}P NMR spectrum of **DM301** in DMSO-d_6.

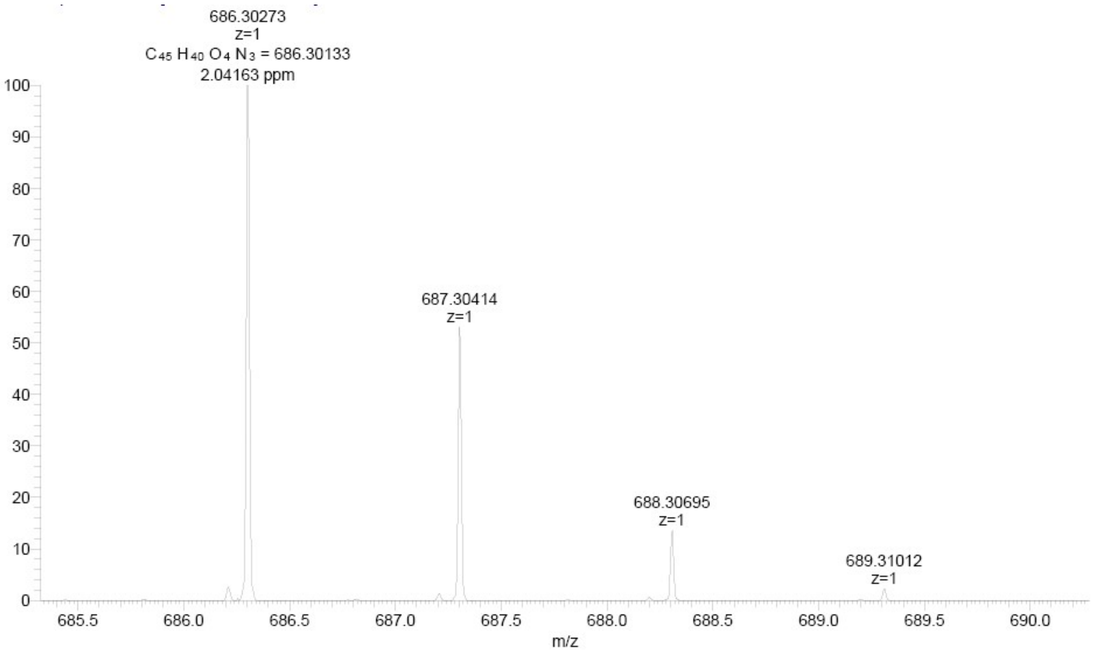

Figure S11. HRMS spectrum of **DM301**

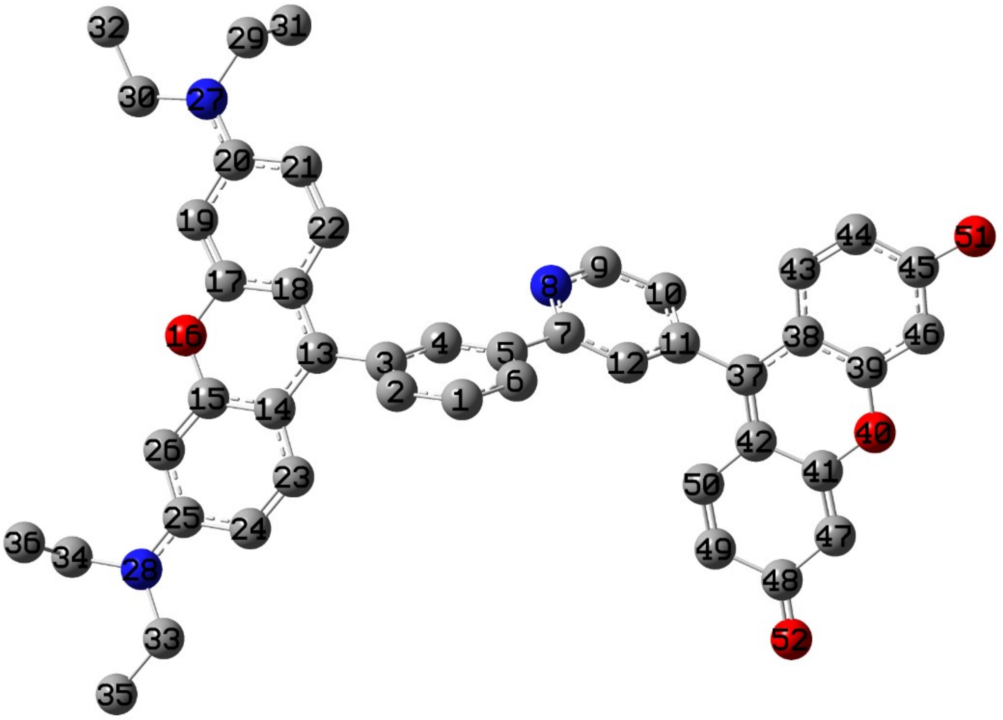

Figure S12. Atoms with labels from optimized structure of **DM301**.

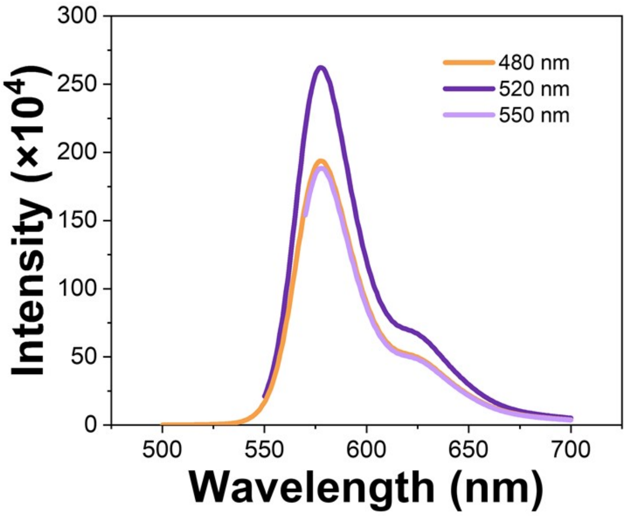

Figure S13. Fluorescence emission of **DM301** under the excitation of lasers of different wavelengths.

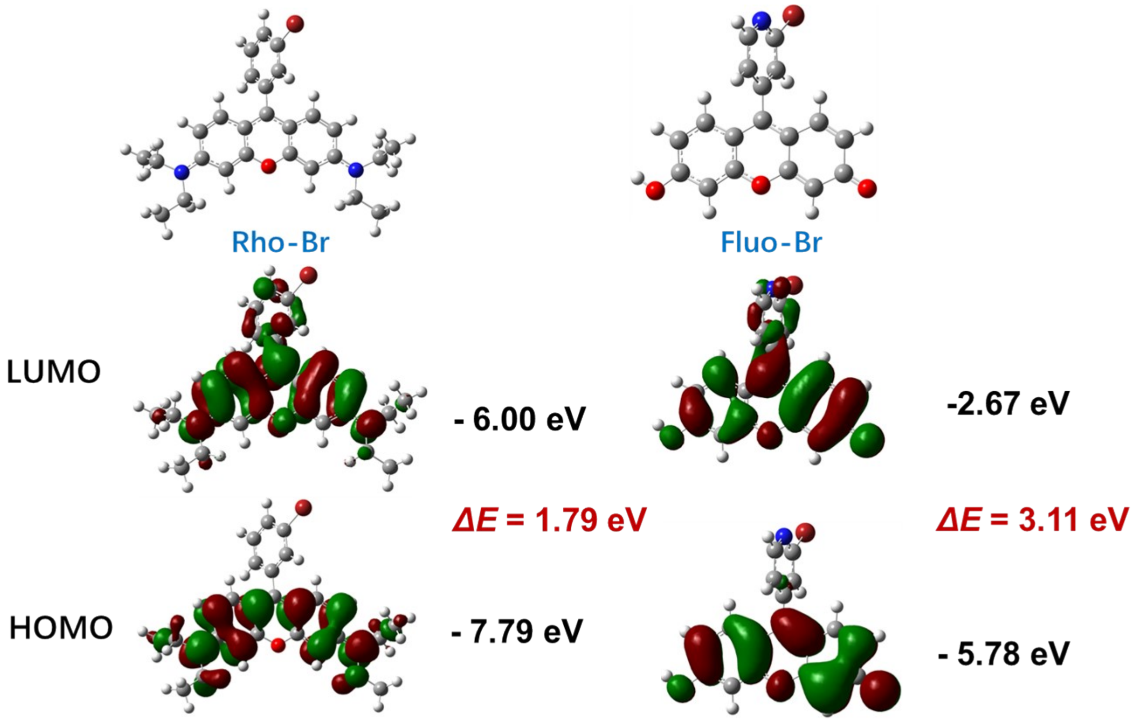

Figure S14. The optimized structure and frontier molecular orbitals of Rho-Br and Fluo-Br in ground state.

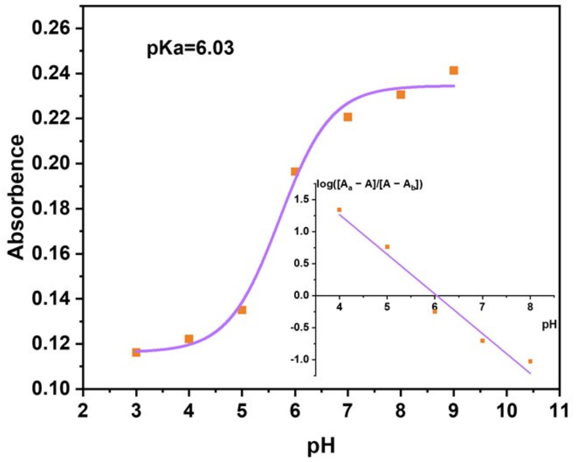

Figure S15. The curve of pH versus absorbance of **DM301** at the absorption wavelength of 510 nm.

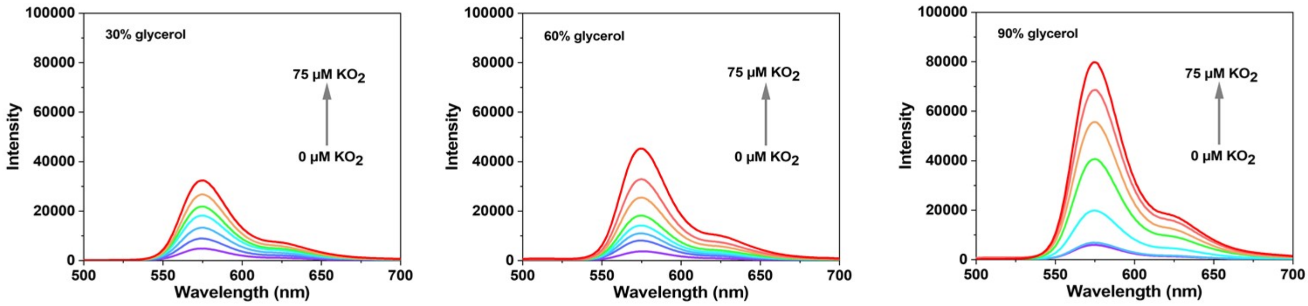

Figure S16. Fluorescence response of DM301 (5 μM) toward KO_2 in aqueous solutions with different viscosity levels (30%, 60%, and 90% glycerol in water, v/v).

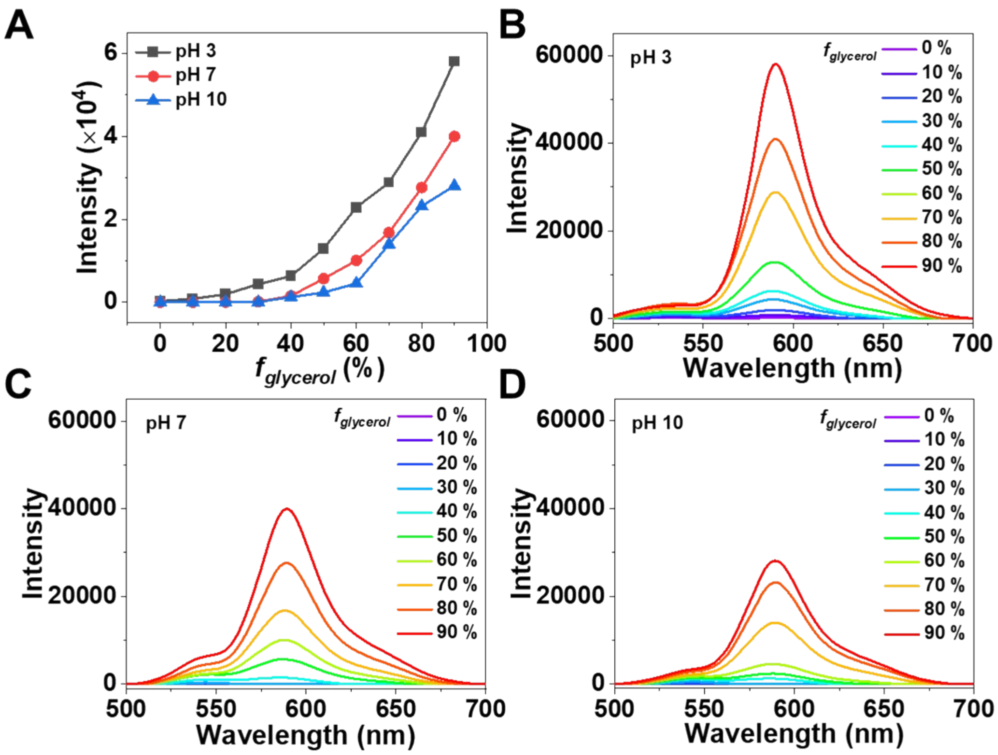

Figure S17. Emission intensity (A) and emission spectra (B, C and D) of **DM301** measured in Britton-Robinson buffer solution (5 μM) with different glycerol percentages under pH 3, 7 and 10, respectively.

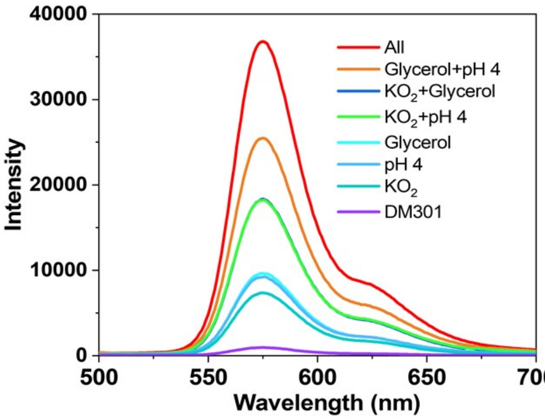

Figure S18. Fluorescence response of DM301 (1 μM) under simultaneous exposure to acidic pH (pH 4), superoxide (35 μM KO_2), and increased viscosity (40% glycerol in water).

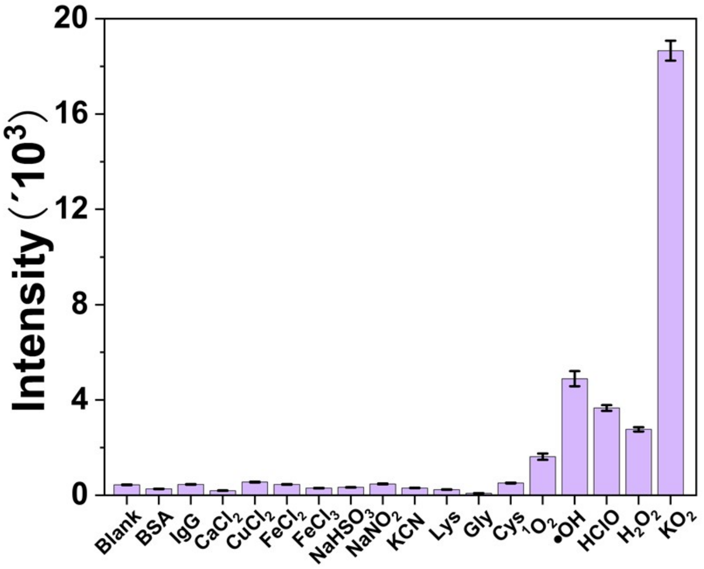

Figure S19. Fluorescence responses of DM301 (50 μM) towards reactive oxygen species ($^1O_2$, H_2O_2, •OH, and KO_2; 100 μM, respectively), proteins (BSA and IgG; 1mg/mL, respectively), metal ions (Ca^{2+}, Cu^{2+}, Fe^{2+}, and Fe^{3+}; 1 mM, respectively), anions (NO_2^-, HSO_3^-, and CN^-; 1 mM, respectively), various amino acids (Lys, Gly, and Cys; 1 mM, respectively), and hypochlorous acid (HClO, 100 μM). All data were acquired in pure water, excited at 480 nm.

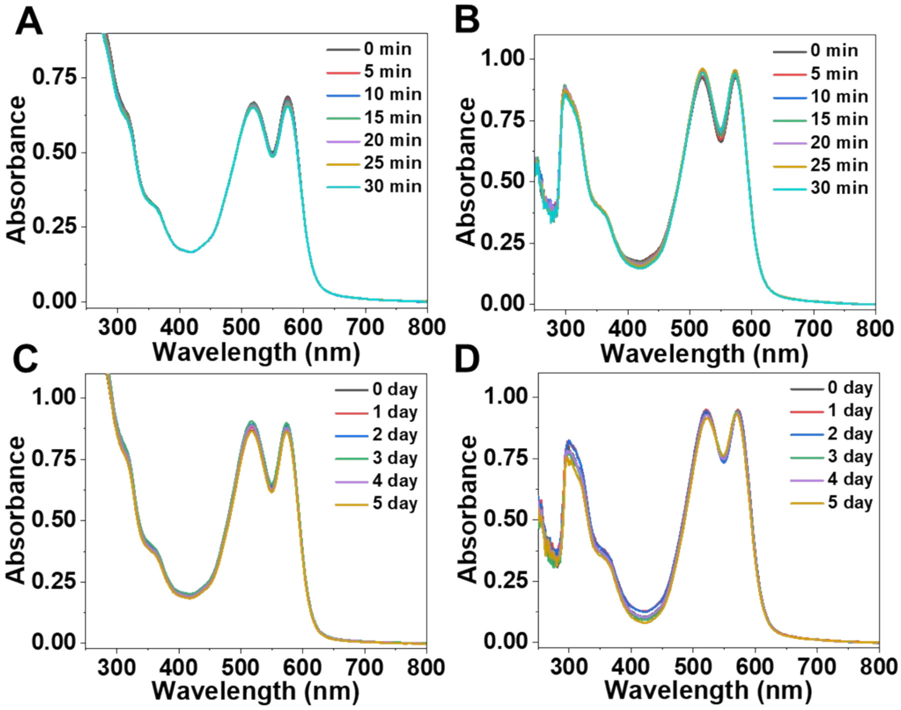

Figure S20. Photostability of **DM301** in (a) PBS and (b) DMEM under continuous white light irradiation (1 W/cm²), as inferred from changes in absorption spectra recorded over 30 minutes at 5-minute intervals. Stability of **DM301** in (c) PBS and (d) DMEM monitored the changes in absorption spectra recorded over 5 days.

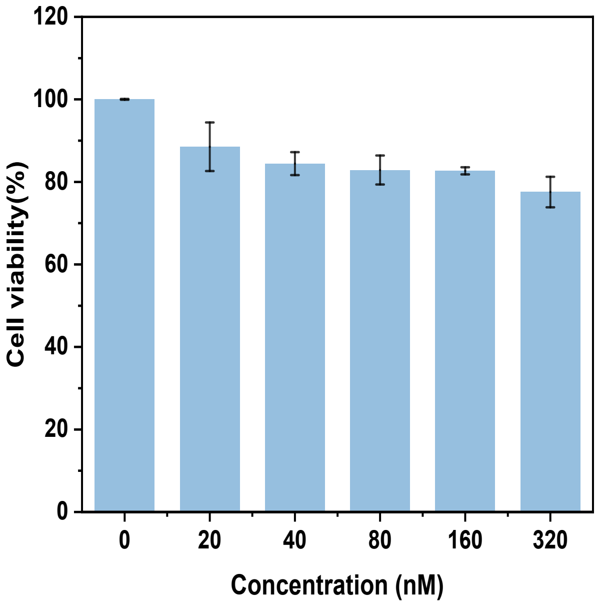

Figure S21. Viability of 3T3 cells after 24 h incubation with various concentrations of DM301.

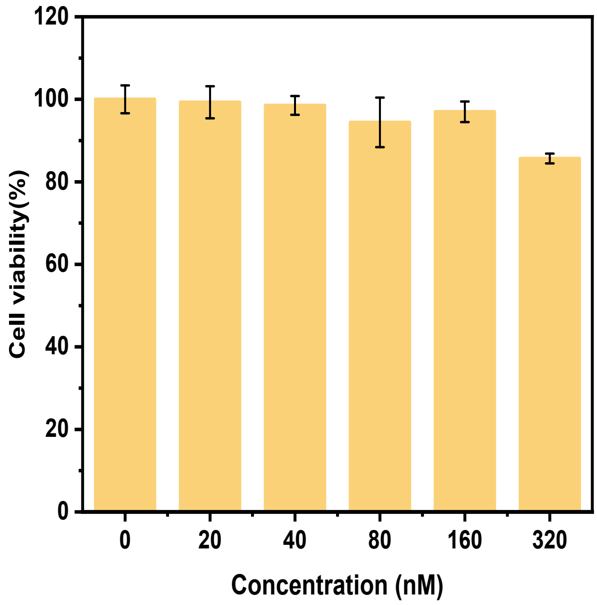

Figure S22. Viability of 4T1 cells after 24 h incubation with various concentrations of DM301.

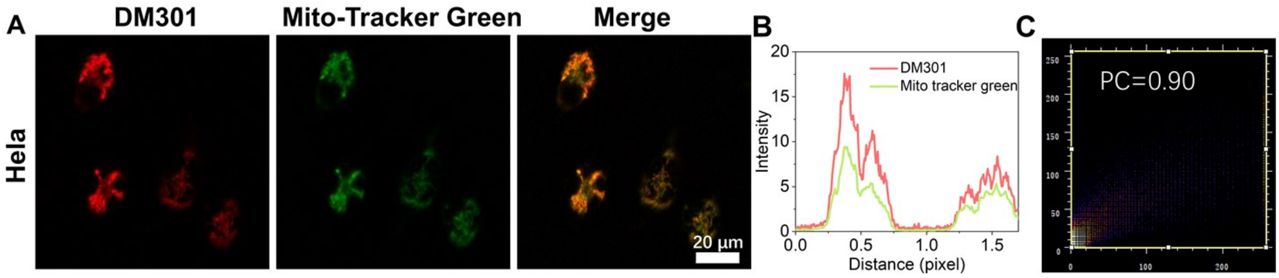

Figure S23. (A) Confocal fluorescence imaging of tumor cells stained with **DM301** (100 nM) and Mito-Tracker Green. (B) Intensity distribution plots and (C) Pearson's coefficients for co-staining of Mito-Tracker Green and **DM301**.

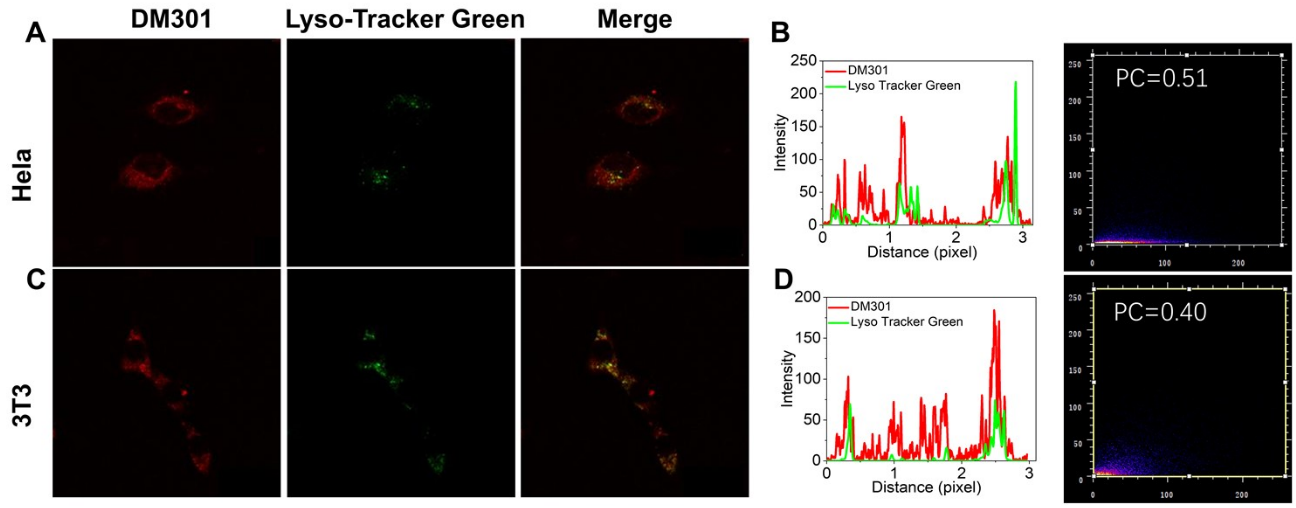

Figure S24. (A) Confocal fluorescence imaging of normal and tumor cells stained with **DM301** (100 nM) and Lyso-Tracker Green. (D) Intensity distribution plots and (E) Pearson's coefficients for co-staining of Lyso-Tracker Green and **DM301**.

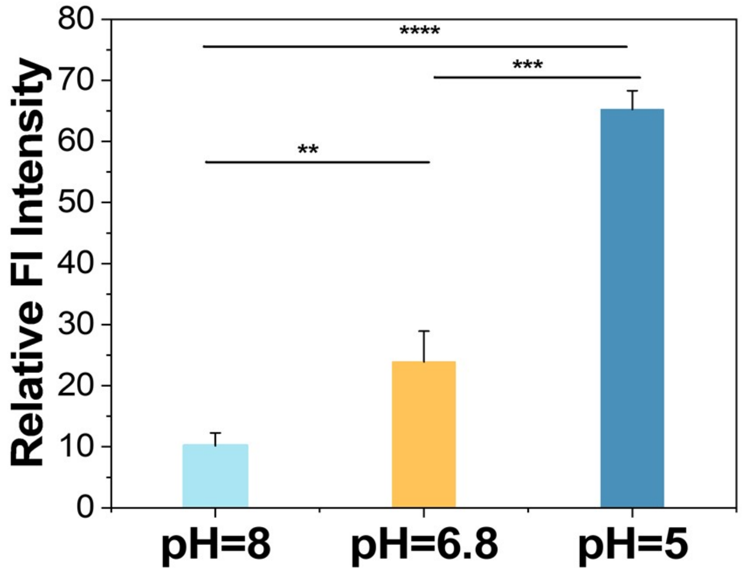

Figure S25. Comparison of tumor cell pH response relative to fluorescence intensity.

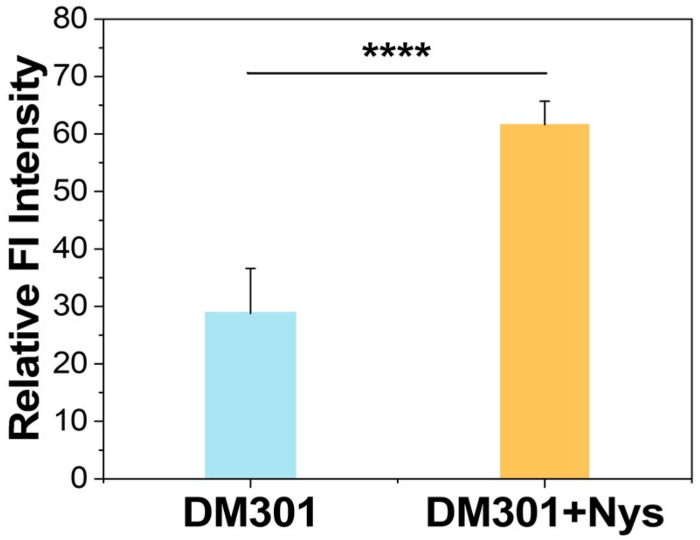

Figure S26. Comparison of relative fluorescence intensity of normal cell viscosity response.

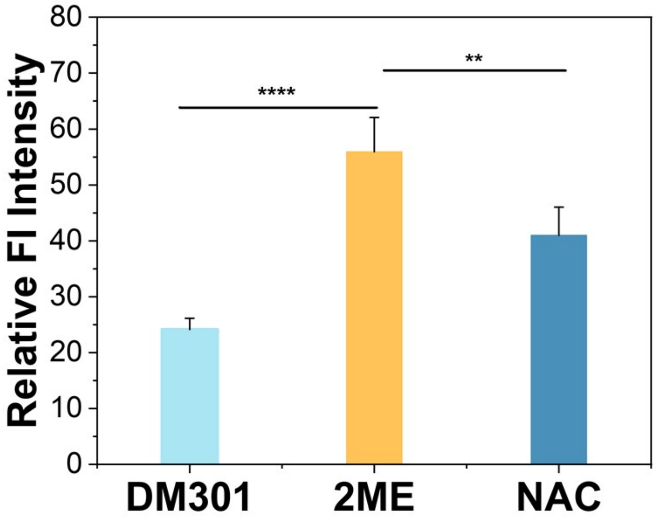

Figure S27. Comparison of $O_2^{\bullet-}$ response relative fluorescence intensity in normal cells.

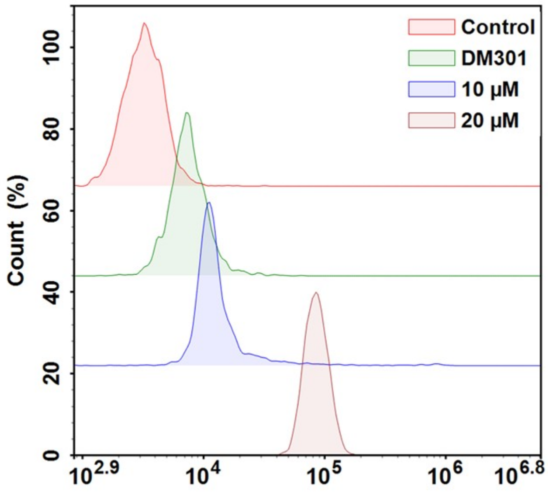

Figure S28. Flow cytometry analysis of AML12 cells incubated with DM301 (50 nM) in the presence or absence of 2-mercaptoethanol (2-ME, 10 μM and 20 μM).

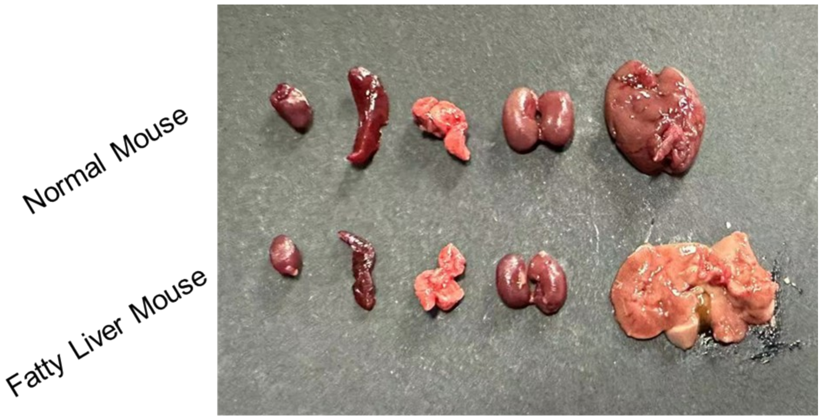

Figure S29. Bright-filed images of organs from normal mice and mice with fatty liver. (**DM301**: 50 μM)

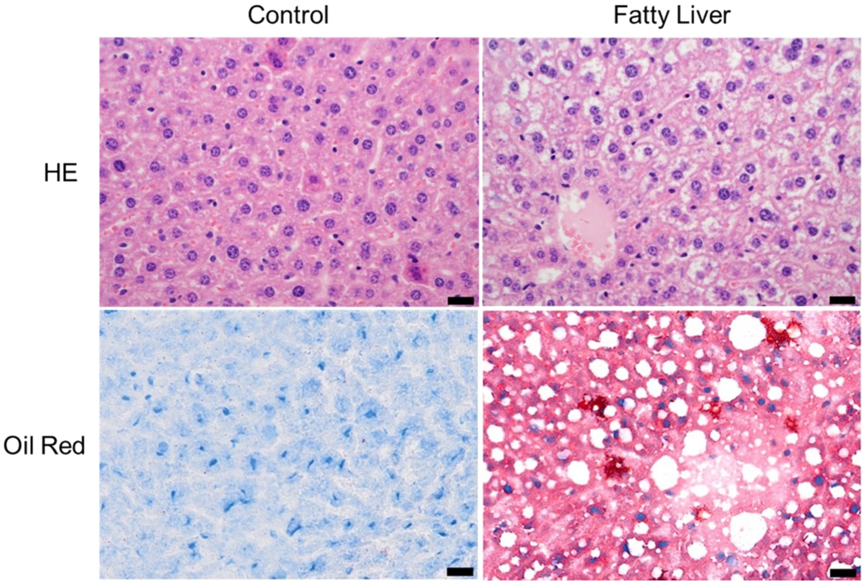

Figure S30. H&E and Oil Red staining of liver from mice with fatty liver. Scale bar: 20 μm.

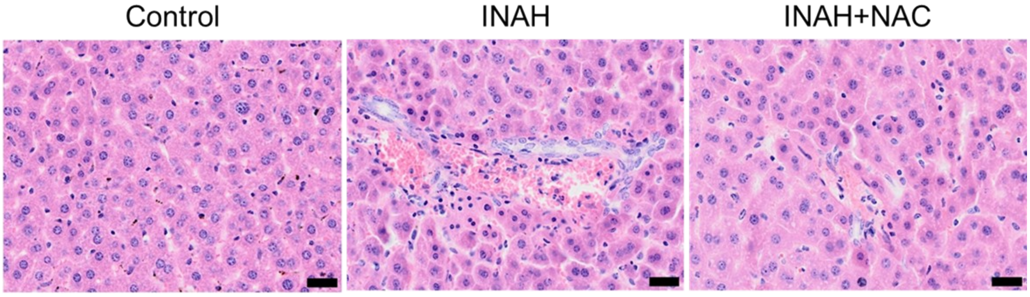

Figure S31. Representative H&E-stained liver tissue sections from mice subjected to different treatments... Scale bar: 20 μm.

Tables

Table S1. UV absorption, molar extinction coefficient and fluorescence emission of **DM301**, Rho-Br, and **DM301**.

Probe	Abs (nm)	ε (*10^4)	Em (nm)
**DM301**	561	4.75	580
**Rho-Br**	562	4.45	583
**Fluo-Br**	463	3.17	541

Table S2. The fluorescence quantum yield (Φf) of the probe in different solutions.

Solvent	DCM	THF	DMSO	DMF	MeCN	MeOH	EG	PBS	Glycerol

**Φ_f**	0.043	0.018	0.009	0.003	0.005	0.008	0.019	0.001	0.104
